# Understanding Sex Differences in Autoimmune Diseases: Immunologic Mechanisms

**DOI:** 10.3390/ijms26157101

**Published:** 2025-07-23

**Authors:** Yu Rin Kim, YunJae Jung, Insug Kang, Eui-Ju Yeo

**Affiliations:** 1 Department of Biomedical Sciences, Graduate School, Kyung Hee University, Seoul 02447, Republic of Korea; kimur1206@naver.com; 2 Biomedical Science Institute, Kyung Hee University, Seoul 02447, Republic of Korea; 3 Department of Microbiology, College of Medicine, Lee Gil Ya Cancer and Diabetes Institute, Gachon University, Incheon 21999, Republic of Korea; yjjung@gachon.ac.kr; 4 Department of Biochemistry, College of Medicine, Gachon University, Incheon 21999, Republic of Korea

**Keywords:** autoimmune diseases, epigenetic regulation, estrogens, immune responses, inflammation, Sjögren’s syndrome, systemic lupus erythematosus, X chromosome inactivation

## Abstract

Autoimmune diseases such as systemic lupus erythematosus and Sjögren’s syndrome show pronounced sex disparities in prevalence, severity, and clinical outcomes, with females disproportionately affected. Emerging evidence highlights sex-based differences in immune and inflammatory responses as key contributors to this bias. Genetic factors—including sex chromosomes, skewed X chromosome inactivation, and sex-biased microRNAs—as well as sex hormones and pregnancy modulate gene expression and immune cell function in a sex-specific manner. Additionally, sex hormone-dependent epigenetic modifications influence the transcription of critical immune regulators. These genetic and hormonal factors collectively shape the activation, differentiation, and effector functions of diverse immune cell types. Environmental factors—including infections, gut microbiota, environmental chemicals and pollutants, and lifestyle behaviors such as diet, smoking, UV exposure, alcohol and caffeine intake, physical activity, and circadian rhythms—further modulate immune function and autoimmune disease pathogenesis in a sex-dependent manner. Together, these mechanisms contribute to the heightened risk and distinct clinical features of autoimmunity in females. A deeper understanding of sex-biased immune regulation will facilitate the identification of novel biomarkers, enable patient stratification, and inform the development of sex-specific diagnostic and therapeutic strategies for autoimmune diseases.

## 1. Introduction

The immune system defends the host against harmful pathogens through tightly regulated inflammatory responses essential for tissue repair and homeostasis maintenance. However, the dysregulation of these pathways can result in persistent inflammation and the breakdown of self-tolerance, ultimately leading to autoimmune diseases [1]. These conditions emerge when the immune system mistakenly targets self-antigens in various tissues—including skin, joints, endocrine organs, and the nervous system—resulting in diverse and often debilitating clinical manifestations [2].

Over 100 autoimmune diseases have been identified, including systemic lupus erythematosus (SLE), Sjögren’s syndrome (SS), rheumatoid arthritis (RA), scleroderma (systemic sclerosis, SD/SSc), multiple sclerosis (MS), type 1 diabetes mellitus, and inflammatory bowel disease. These diseases are chronic, often lifelong, and significantly impair quality of life, increase morbidity, and pose substantial socioeconomic burdens. Their global incidence and prevalence continue to rise, with an estimated 23.5 million individuals affected in the United States alone—approximately 10% of the global population. Prevalence varies according to genetic background, geographic location, environmental exposures, and coexisting conditions, emphasizing the need for nuanced epidemiological insights to inform public health interventions and precision medicine approaches [3].

**Figure 1:**
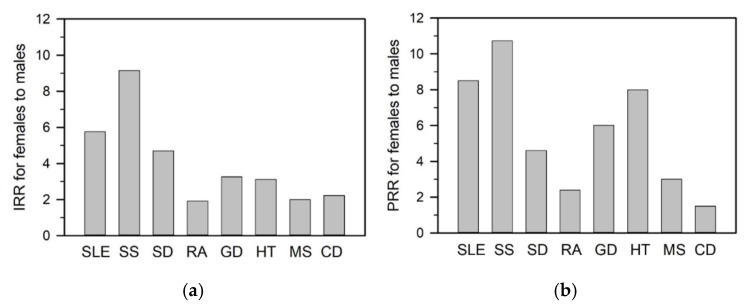
The incidence rate ratio (IRR) and prevalence rate ratio (PRR) for females compared to males across various autoimmune diseases. The IRR (**a**) and PRR (**b**) are derived from referenced epidemiological data for each disease: systemic lupus erythematosus (SLE) [7,8,9], Sjögren’s syndrome (SS) [6,10], scleroderma (SD) [11,12], rheumatoid arthritis (RA) [13,14,15], Graves’ disease (GD) [3,16], Hashimoto’s thyroiditis (HT) [3,17], multiple sclerosis (MS) [18,19], celiac disease (CD) [20,21]. These ratios highlight the striking sex disparities in disease incidence and prevalence across autoimmune conditions.

A defining feature of autoimmune diseases is their marked sex disparity [4,5]. Female-to-male incidence and prevalence ratios vary across diseases and populations, with the greatest skew observed in SLE and SS [3,6,7,8]. SLE shows incidence and prevalence rate ratios of 5.8 and 8.5, respectively, while SS exhibits even higher ratios of 9.2 and 10.7 (Figure 1a,b). Given these pronounced differences, this review highlights SLE and SS as representative models of female-biased autoimmunity.

SLE is a prototypic systemic autoimmune disorder characterized by multisystem involvement—including the skin, joints, kidneys, central nervous system, lungs, and vasculature. Its clinical presentation is heterogeneous, with common symptoms such as fatigue, fever, cytopenia, arthritis, malar rash, and proteinuria [22]. Lupus nephritis (LN), a major complication, occurs in ~40% of patients and progresses to end-stage renal disease in approximately 10% within a decade [23,24,25]. LN is more prevalent in females and peaks in incidence between the ages 30–39, then declines after age 60 [26,27].

SS is a chronic autoimmune disease that predominantly affects the exocrine glands, particularly the lacrimal and salivary glands, resulting in dryness of the eyes (xerophthalmia) and mouth (xerostomia). It can occur as primary SS (pSS) or as secondary SS in association with other autoimmune diseases such as SLE, RA, or SD. SS is characterized by lymphocytic infiltration of the glands, reduced secretory capacity, and systemic complications, including interstitial lung disease, cardiovascular manifestations, and renal dysfunction [28,29].

Biological sex and sex hormones are critical determinants of immune responses to pathogens and self-antigens [30,31]. Females generally exhibit stronger innate and adaptive immune responses than males, enhancing pathogen defense but increasing susceptibility to autoimmunity [2,32]. Section 2 of this review summarizes the key methodologies used in the field, including sex-stratified cellular assays, genetically and hormonally manipulated animal models, clinical cohort and epidemiological studies, and multi-omics and systems biology approaches. Section 3 outlines immunological disparities between sexes, while Section 4 explores how these differences contribute to sex-biased susceptibility, onset, and progression of SLE and SS. The heightened autoimmune risk observed in females is attributed to X chromosome-linked gene dosage effects and epigenetic regulation, sex hormone-mediated modulation of immune pathways, and environmental factors [33]. Section 5 explores these genetic and hormonal mechanisms and environmental insults underlying sex bias in autoimmunity. Understanding these mechanisms is essential to advance our knowledge of autoimmune pathogenesis and to guide the development of sex-informed, personalized therapeutic strategies.

## 2. Experimental Approaches to Investigating Sex Differences in Autoimmunity

The female predominance in SLE and pSS underscores the impact of biological sex on disease susceptibility and progression [33]. To delineate the mechanisms driving these sex differences, diverse experimental strategies have been employed, spanning cellular and molecular analyses, genetically and hormonally manipulated animal models, clinical cohort studies, and integrative multi-omics approaches. A summary of these methodologies follows.

### 2.1. Cellular and Molecular Studies

Sex-stratified immune cell assays are pivotal for identifying intrinsic cellular differences. Primary immune cells, such as monocytes, plasmacytoid dendritic cells (pDCs), T cells, and B cells, are isolated from male and female donors and assessed for functional parameters such as cytokine production, proliferation, apoptosis, and signaling pathways [32,34,35].

Hormonal modulation studies using estradiol, testosterone, or progesterone evaluate the impact of sex steroids on immune cell phenotypes, gene expression, and signaling cascades [36,37]. Estrogen receptor alpha (ERα)-knockout mouse models further dissect estrogen receptor-mediated effects on regulatory T cells (T_regs_) and inflammatory responses [38].

Epigenetic profiling—through ATAC-seq (chromatin accessibility), ChIP-seq (histone modifications), and DNA methylation analyses—reveals sex-specific chromatin landscapes and X chromosome inactivation (XCI) patterns [39,40]. Functional genomics tools, including CRISPR/Cas9 and RNA interference, target sex-biased genes such as forkhead box P3 (*FoxP3*) and hormone receptors to define their regulatory roles in immune responses [32].

### 2.2. Animal Models with Genetic or Hormonal Manipulations

Animal models offer a controlled platform to dissect the influence of sex chromosomes, hormones, and gene expression. Female-biased disease phenotypes are observed in lupus-prone strains such as MRL/lpr, NZB/NZW F1, and NZM2410 [38]. Key strategies include:

Gene knockout or transgenic models: ERα-deficient mice, Toll-like receptor 7 (*TLR7*)-overexpressing strains, and *FoxP3*-GFP knock-ins elucidate hormone and gene dosage effects [41,42,43].

Hormonal manipulation: Gonadectomy (e.g., ovariectomy or orchiectomy) and subsequent hormone replacement clarify the immune-modulatory roles of estrogen and testosterone [44].

Bone marrow chimeras: Transplantation of bone marrow from male or female donors into sex-matched or mismatched recipients allows for the dissection of hematopoietic versus non-hematopoietic (stromal) contributions to sex-specific immune regulation [45].

X-chromosome dosage models: Four Core Genotype (FCG) mice decouple chromosomal from gonadal sex effects [46,47].

SS-like pathology develops in aromatase-knockout and NOD-derived mice, which serve as specialized models for pSS [48]. Sex-specific immune infiltration and organ pathology are visualized using immunohistochemistry and in vivo imaging techniques (e.g., PET, bioluminescence) [49,50].

### 2.3. Clinical and Epidemiological Studies

Sex-stratified clinical cohort studies illuminate real-world disease expression, revealing differences in incidence, severity, autoantibody profiles, and treatment responses [51]. Longitudinal designs link immune biomarkers to hormonal milestones (e.g., menstrual cycle, pregnancy, menopause, hormone replacement therapy) [48].

Genetic studies—including Genome-Wide Association Study (GWAS) and a multilayered post-GWAS analyses—have identified sex-biased risk loci such as human leukocyte antigen (HLA), *TLR7*, interferon regulatory factor (*IRF5*), and lysine demethylase 6A (*KDM6A*) [52,53,54]. Additionally, sex-based subgroup analyses in clinical trials reveal divergent therapeutic responses and toxicity profiles [55,56].

Studies of individuals with sex chromosome aneuploidies (e.g., 45,X; 47,XXY) provide further insight into gene dosage versus hormonal influences on autoimmunity. Epidemiological analyses also explore sex-specific environmental exposures and familial patterns.

### 2.4. Multi-Omics, Systems Biology, and Computational Modeling

High-throughput omics technologies are central to unraveling sex-biased immune regulation. Bulk and single-cell RNA-seq, proteomics, and metabolomics delineate transcriptional, translational, and metabolic differences in immune cells from male and female patients or animal models [32,57,58].

Sex-differential expression of non-coding RNAs (e.g., miR-155, Xist) further refines the molecular landscape [59,60]. Microbiome analyses uncover sex-dependent interactions between gut microbes and immune regulation [61].

Computational approaches integrate omic and clinical data to construct sex-specific regulatory networks. Machine learning algorithms and network-based analyses identify sex-biased pathways and predict disease phenotypes, offering a framework for sex-informed precision medicine in autoimmunity [40].

## 3. Sex-Specific Disparities in Immune Responses

The immune system functions through tightly regulated innate and adaptive mechanisms. Innate immunity provides rapid, nonspecific defense via myeloid cells and TLRs, which activate proinflammatory and interferon (IFN) pathways. Adaptive immunity, driven by antigen-specific T and B cells, enables long-term protection but may promote autoimmunity when tolerance fails. Key processes—including T cell differentiation, B cell maturation, cytokine production, and antigen presentation—are influenced by sex-linked factors such as hormones, epigenetic regulation, and X chromosome gene dosage. This section outlines these core immune mechanisms to establish a framework for examining how biological sex shapes immune function and autoimmune risk.

### 3.1. Innate Immune Responses

**Figure 2:**
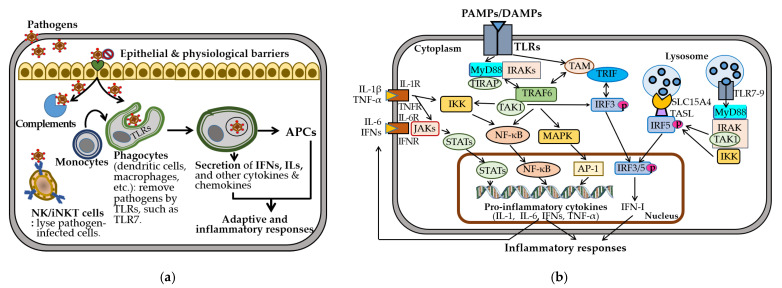
Innate immune responses. (**a**) Innate immunity involves epithelial and physiological barriers, complement activation, and recruitment of innate immune cells. TLRs initiate phagocytosis and stimulate the production of interferons (IFNs), interleukins (ILs), and other cytokines and chemokines, facilitating antigen presentation through the generation of antigen-presenting cells (APCs). (**b**) Innate immune responses triggered by pathogen-associated molecular patterns (PAMPs) or damage-associated molecular patterns (DAMPs) initiate inflammation, primarily via surface TLR-mediated signaling through the Myddosome complex (MyD88 and IRAKs), leading to the activation of transforming growth factor-β-activated kinase 1 (TAK1), NF-κB, MAPK, and interferon regulatory factor (IRF) pathways. These cascades promote the nuclear translocation of NF-κB, AP-1, and IRF3/5, driving proinflammatory cytokine expression. Endosomal TLR7-9 and TLR4 also activate IRF5 via the SLC15A4-associated adaptor TASL, enhancing type I interferon (IFN-I) production. These cytokines further amplify inflammation by activating downstream Janus kinase-signal transducers and the activator of transcription (JAK-STAT), NF-κB, and MAPK-AP-1 signaling pathways through their respective receptors. All other abbreviations are defined in the main text and listed at the end of the manuscripts. All one-way arrows indicate activation and forward progression along the pathway, while bi-directional arrows represent interactions between components.

The innate immune system provides rapid, nonspecific defense through physical barriers, innate immune cells—including dendritic cells (DCs), macrophages, natural killer (NK) cells, and invariant natural killer T (iNKT) cells—and soluble mediators such as cytokines and chemokines (Figure 2a). These responses enhance antigen presentation by promoting the generation of antigen-presenting cells (APCs) and driving adaptive and inflammatory responses.

TLRs play a central role in recognizing pathogen-associated and damage-associated molecular patterns and in mediating cell-based immunity [62]. Surface TLRs (e.g., TLR1/2, TLR4-6, TLR10) detect microbial components, while endosomal TLRs (e.g., TLR3, TLR7-9) recognize nucleic acids from viruses, bacteria, or self-origin [63]. Upon ligand binding, TLRs assemble the Myddosome complex, comprising myeloid differentiation factor 88 (MyD88) and interleukin 1 receptor-associated kinases (IRAKs) [64]. This complex activates transforming growth factor- β-activated kinase 1 (TAK1), nuclear factor kappa B (NF-κB), mitogen-activated protein kinase (MAPK), and IRF pathways [63] (Figure 2b). TLR3/7/8/9 are trafficked from the endoplasmic reticulum to endosomes by UNC93B1, an endoplasmic transmembrane protein highly expressed in DCs, macrophages, monocytes, and B cells. Endosomal TLR7-9 activate IRF5 via TASL, a TLR adaptor that interacts with the endolysosomal solute carrier family 15 member 4 (SLC15A4) [65,66]. This signaling induces proinflammatory cytokines, including interleukins (IL-1β, IL-6), tumor necrosis factor-alpha (TNF-α), and type I interferons (IFN-Is), which are crucial for pathogen clearance but contribute to autoimmunity when dysregulated [67,68,69].

Sex-specific differences shape these immune responses [31,70]. Females exhibit higher phagocyte numbers, TLR expression, and production of IL-6 and TNF-α, promoting stronger innate activation. Although females have a lower percentage of NK cells, males exhibit greater NK cell-mediated cytotoxicity and more robust anti-inflammatory response. These differences may underlie the higher autoimmune susceptibility observed in females.

Collectively, the innate immune system initiates rapid, nonspecific responses through physical barriers, immune cells (e.g., DCs, macrophages, NK cells), and soluble mediators like cytokines. TLRs are central to pathogen sensing, with endosomal TLR7–9 activating IRF5 and IFN-I pathways via MyD88, UNC93B1, and TASL–SLC15A4 complexes. While critical for pathogen clearance, dysregulation of these pathways contributes to autoimmunity. Notably, sex-based differences influence innate immunity: females exhibit higher TLR expression and cytokine production, while males show greater NK cell cytotoxicity. These differences may contribute to the heightened autoimmune risk observed in females.

### 3.2. Adaptive Immune Responses

**Figure 3:**
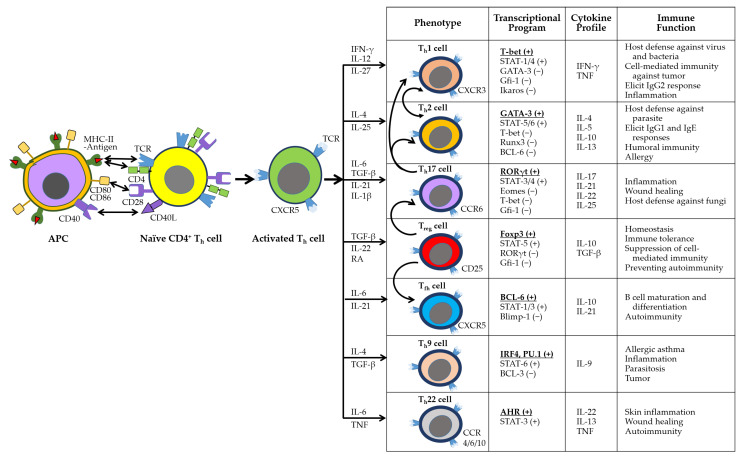
The activation and differentiation of naïve CD4^+^ T_h_ cells. Cytokines produced by activated phagocytes and T_h_ cells guide the differentiation of naïve CD4^+^ T_h_ cells into distinct subsets, including T_h_1, T_h_2, T_h_17, T_reg_, T_fh_, T_h_9, and T_h_22. Each subset is characterized by specific phenotypes, transcriptional programs, cytokine profiles, and immune functions. The subsets also exhibit functional plasticity: T_h_1 and T_h_2 cells can interconvert, while T_h_17 and T_reg_ cells are unstable and may transition into other lineages, as indicated by curved arrows in the phenotype panel. In the transcriptional profile panel, activating and inhibitory transcription factors are marked by (+) and (−), respectively, with the master regulator listed first and underlined.

Adaptive immunity is mediated by antigen-specific responses of T and B cells. Upon antigen presentation via major histocompatibility complex class II (MHC-II) on APCs, naïve CD4^+^ T cells differentiate into specialized helper T cell subsets (e.g., T_h_1, T_h_2, T_h_17, T_reg_, T_fh_, T_h_9, T_h_22) to orchestrate immune responses (Figure 3). These subsets exhibit distinct transcriptional programs, cytokine secretion profiles, and immunological functions. Notably, T_h_1, T_h_9, T_h_17, and T_h_22 cells promote inflammation and contribute to autoimmunity [71,72].

**Figure 4:**
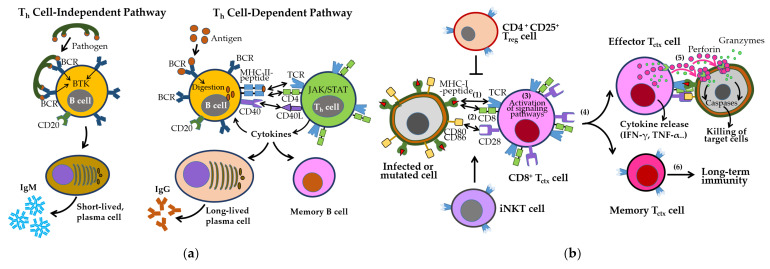
The activation and differentiation of B cells and CD8^+^ T_ctx_ cells. (**a**) B cell activation in humoral immunity. B cells can be activated independently of T_h_ cells by pathogens displaying repetitive epitopes, leading to B cell receptor (BCR) crosslinking and differentiation into short-lived plasma cells that secrete IgM antibodies. Alternatively, BCR-mediated antigen uptake followed by MHC-II presentation to T_h_ cells induces signaling cascades that promote the generation of long-lived plasma cells and memory B cells. This results in high affinity IgG production and robust humoral immunity. (**b**) CD8^+^ T_ctx_ cell activation in cell-mediated immunity. CD8^+^ T_ctx_ cells recognize infected or transformed cells through T cell receptor (TCR)-MHC-I-peptide interactions (step 1), along with co-stimulatory signals via CD28 and CD80/CD86 (step 2). This triggers activation of signaling pathways (step 3) and differentiation into effector and memory T_ctx_ cells (step 4). Effector cells secrete cytokines and cytotoxic molecules such as perforin and granzymes, enabling the elimination of target cells (step 5). Memory T cells help provide long-term protection by quickly responding to a previously encountered antigen (step 6). These responses are modulated by T_reg_ and iNKT cells. All one-way arrows indicate activation and forward progression along the pathway, bidirectional arrows represent interactions between components, and truncated lines indicate inhibition.

B cells are activated either independently or with T_h_ cell help, undergoing class switching and affinity maturation in germinal centers [73,74]. These processes generate short-lived IgM-secreting plasma cells, long-lived IgG-secreting plasma cells, and memory B cells (Figure 4a). B cell survival and differentiation are regulated by B cell activating factor (BAFF) and a proliferation-inducing ligand (APRIL) via signaling through BAFF receptor (BAFFR), transmembrane activator and calcium moderator and cyclophilin ligand interactor (TACI), and B cell maturation antigen (BCMA) [75].

CD8^+^ cytotoxic T cell (T_ctx_), activated by MHC-I–presented antigens and costimulatory signals (e.g., CD28–CD80/CD86), eliminate infected or abnormal cells through perforin/granzyme release and proinflammatory cytokine production (Figure 4b). Their cytotoxic activity is tightly regulated by inhibitory checkpoints, including T_ctx_-associated protein 4 (CTLA-4), PD-1/PD-L1, TIM-3, and VISTA [76,77], as well as T_reg_-mediated suppression. iNKT cells further support memory T cell formation and cross-priming [78].

During immune maturation, self-reactive immune cells are eliminated through central tolerance [79]. However, this process is incomplete and reinforced by peripheral tolerance mechanisms, including the conversion of self-reactive T_h_ cells into T_regs_ [80]. In autoimmune diseases, T_reg_ cells are often reduced in number, exhibit impaired suppressive function, or both. The presence of autoantibodies and autoreactive T cells, along with decreased T_reg_ populations, is a hallmark of autoimmunity [81,82].

Sex-specific differences further shape adaptive immune responses. In the humoral compartment, females exhibit higher B cell counts and a greater propensity for differentiation into autoantibody-producing plasma cells compared with males [70]. These autoantibodies activate the classical complement pathway, perpetuating inflammation and tissue damage [83]. Elevated BAFF/APRIL activity in females may further reinforce these responses, contributing to the higher severity and mortality of autoimmune diseases [70].

Females also display heightened lymphocyte activation, increased CD4^+^ T_h_ cell counts, elevated cytokine production (e.g., ILs, IFNs), and a higher CD4^+^/CD8^+^ T cell ratio, resulting in stronger—yet often pathological—immune responses [70,84]. In contrast, males exhibit a predominance of T_reg_ cells, contributing to weaker immune responses [85]. T_h_17-skewed inflammation is more prominent in females and is closely associated with autoimmune diseases such as SLE and RA. Additionally, sex-specific differences in APC function, including MHC-II usage, modulate T cell receptor (TCR) signaling thresholds—the minimum level of TCR stimulation required to trigger downstream signaling events that determine T cell activation, differentiation, and the induction of immune tolerance [86]. Overexpression of X-linked immune genes (e.g., *CD40L*, *CXCR3*, *OGT*) in females further amplifies immune responses.

In summary, adaptive immunity relies on antigen-specific responses of T and B cells, with T_h_1, T_h_17, T_fh_, and activated B cells playing central roles in inflammation and autoantibody production. Autoimmune pathology involves impaired tolerance mechanisms and dysregulated effector responses, including reduced T_reg_ function and enhanced T_h_17 activity. Sex-based differences contribute significantly: females exhibit higher B cell counts, elevated BAFF/APRIL signaling, stronger CD4^+^ T cell responses, and greater autoantibody production, while males show increased T_reg_ activity. These disparities are further shaped by differential APC function and overexpression of X-linked immune genes, promoting heightened immune activation in females and increased susceptibility to autoimmune diseases.

## 4. Alterations of Immune and Inflammatory Responses in SLE and SS

In both SLE and SS, IFN-I signaling, initiated by TLRs and sustained by pDCs and immune complexes, drives proinflammatory cytokine production and autoantibody generation, leading to tissue inflammation and organ damage. In SLE, TLR7 overactivation, impaired immune regulation, and dysfunctional B–T cell interactions promote chronic inflammation. B cells function as both antibody producers and APCs, while CD8^+^ T_ctx_ cells show reduced cytotoxicity. Similarly, in SS, IFN-driven innate activation progresses to adaptive immune responses, characterized by B cell hyperactivity and T_h_1/T_h_17 polarization, promoting autoantibody production and glandular infiltration. Epithelial cells and CD8^+^ T_ctx_ further contribute to local inflammation and tissue damage. Sex differences are prominent in both diseases: females exhibit stronger humoral responses, whereas males more often develop severe organ involvement. This section examines the sex-specific immune and inflammatory alterations underlying SLE and SS pathogenesis.

### 4.1. Immune and Inflammatory Responses Manifested in SLE

**Figure 5:**
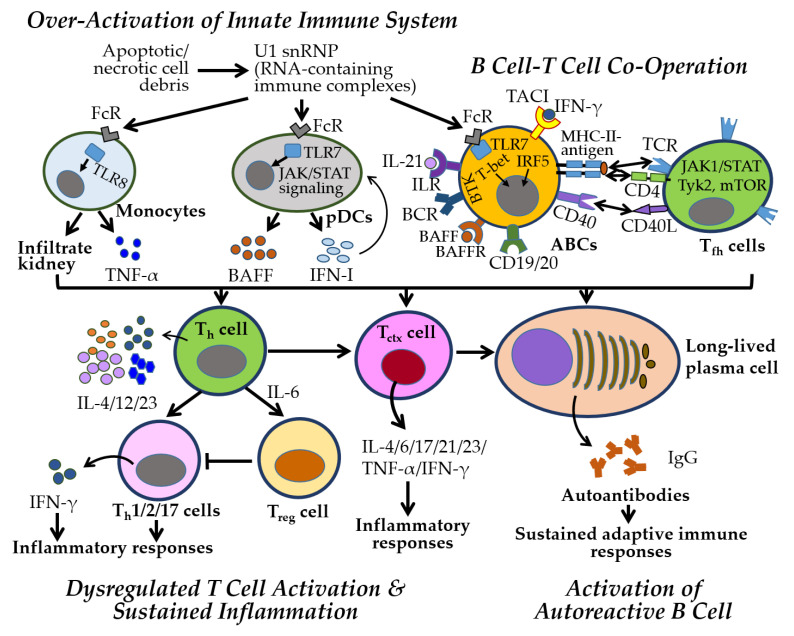
Key immune and inflammatory responses in SLE. Innate immunity plays a central role in the early stages of SLE through the activation of the IFN-I pathway. In SLE, U1 snRNP (small nuclear ribonucleoprotein, RNA-containing immune complexes), derived from apoptotic or necrotic cell debris, are taken up by immune cells via Fc receptors (FcRs), and the single-stranded RNA within U1 snRNP is recognized by endosomal TLRs. This recognition triggers IFN-I production by plasmacytoid dendritic cells (pDCs) via the JAK-STAT pathway. TLR7 activation promotes the differentiation of age-associated B cells (ABCs) into autoantibody-producing cells, while TLR8 activation stimulates TNF-α-producing monocytes that infiltrate the kidney. B cells, activated through BCR engagement and receptors such as BAFFR, TACI, APRIL, and CD19/CD20, interact with T cells through MHC-II-antigen–TCR/CD4 and CD40–CD40L signaling. Cytokines such as IL-4, IL-12, and IL-23 further promote T cell activation and inflammation. Autoantibodies form immune complexes that perpetuate adaptive immune responses and drive disease progression. All other abbreviations are defined in the main text and listed at the end of the manuscript. All one-way arrows indicate activation and forward progression along the pathway, bidirectional arrows represent interactions between components, and truncated lines indicate inhibition.

SLE pathogenesis involves innate and adaptive immune activation, cytokine dysregulation, autoantibody production, and immune complex deposition, ultimately driving chronic inflammation and organ damage [25,87] (Figure 5). A hallmark feature is the production of antinuclear antibodies against double-stranded DNA (anti-dsDNA) and small nuclear ribonucleoproteins (snRNPs), such as Smith protein (Sm) and Sjogren’s autoantibodies (SSA, and SSB) [69]. However, the data regarding their effectiveness as a predictive marker for LN is not fully established [24]. Upregulation of IFN-stimulated genes (ISGs), transcription factors (e.g., STAT3, TASL), and proinflammatory cytokines (e.g., IL-6, TNF-α) links innate activation to adaptive immune dysregulation, promoting B cell activation, autoantibody production, and tissue inflammation [88,89,90].

Enhanced TLR7 signaling is central to SLE pathogenesis through (1) increased TLR7 expression via gene duplication or stabilizing SNPs, (2) sustained activation by endosomal ligand accumulation, and (3) gain-of-function mutations lowering activation thresholds [47,63,69,91]. In pDCs and myeloid DCs, TLR7 drives IFN-I and cytokine production, and chemokine migration to inflammatory sites, promoting autoimmunity and tissue damage [92]. RNA-containing immune complexes further activate TLR7/8, sustaining inflammation via the JAK–STAT pathway and promoting autoreactive B cell differentiation into autoantibody-producing cells through Bruton’s tyrosine kinase (BTK), BAFFR, TACI, CD19, and CD20 [93].

B–T cell interactions amplify adaptive immune dysregulation. CD40–CD40L engagement promotes B cell activation, class switching, and production of IgA, IgG, and IgM autoantibodies, forming immune complexes that deposit in tissues—particularly the kidneys [23,24,94]. B cells also act as APCs, activating autoreactive CD4^+^ T_h_ cells and CD8^+^ T_ctx_, which further sustain inflammation through IFN-I and IFN-II (IFN-γ) production [95,96]. Elevated levels of IFN-III (IFN-λ), which contributes to mucosal immunity via the JAK-STAT pathway, have been detected in the blood and tissues of patients and animal models with autoimmune rheumatic diseases, including SLE and pSS, suggesting a complex role in regulating innate and adaptive immune responses in their pathogenesis [97]. Altered TCR signaling and endocytic recycling in T_h_ cells promote proinflammatory polarization and impair T_reg_ function, contributing to systemic inflammation [82,98]. Genetic, hormonal, and environmental factors exacerbate these immune disturbances.

CD8^+^ T_ctx_ cells in SLE show impaired cytotoxic function despite elevated activation markers (CD38, HLA-DR), contributing to both defective pathogen clearance and autoimmune tissue damage [99]. γδ T cells and IL-15–driven CD4^+^ CD28^−^ T cells also promote tissue injury in LN through antigen presentation and proinflammatory cytokine secretion [100]. T_ctx_ cells targeting modified self-antigens contribute directly to tissue damage [101], while altered immunometabolism, marked by increased glycolysis and oxidative stress, exacerbates inflammation in both T and B cells [102,103,104]. Autoreactive B cells further sustain inflammation by functioning as APCs and producing cytokines. Loss of B cell tolerance is driven by B cell receptor (BCR) and IFN-I signaling from pDCs, while T_fh_ cells promote B cell activation through IL-4, IL-17, IL-21, and IFN-γ [105].

SLE pathogenesis involves well-established mechanisms such as TLR7-driven IFN-I production, B–T cell interactions, and autoantibody formation, leading to chronic inflammation and tissue damage. These processes are consistently supported by clinical and experimental data. Emerging evidence suggests additional roles for IFN-III, altered TCR signaling, and immunometabolic dysregulation in promoting proinflammatory T cell responses and impairing immune tolerance. CD8^+^ T cell dysfunction and γδ T cell–mediated injury in LN are supported by preclinical findings but remain less well-defined clinically. Sex-based differences—characterized by broader immune activation in females and more severe renal involvement in males—are evident and increasingly recognized as critical to disease expression. While several pathways are well-characterized, others remain suggestive and require further validation across model systems.

### 4.2. Immune and Inflammatory Responses Manifested in SS

**Figure 6:**
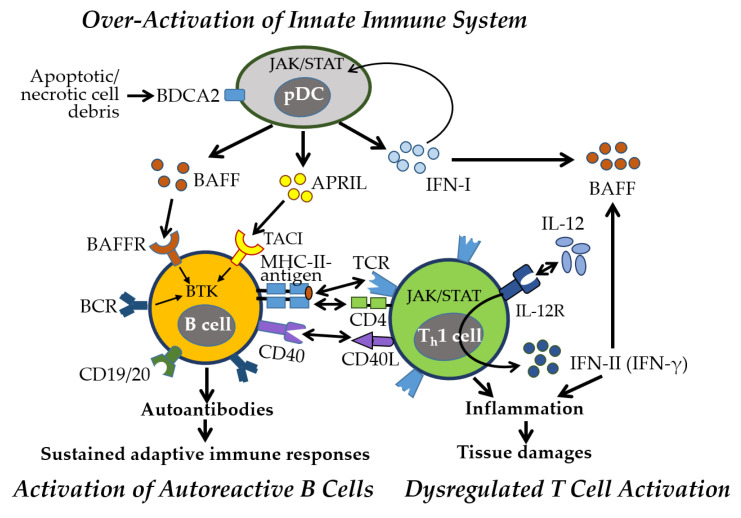
Key immune and inflammatory responses in pSS. Early activation of the innate immune system, particularly the IFN-I pathway, plays a central role in pSS. Activated pDCs by blood dendritic cell antigen 2 (BDCA2) secrete IFN-I and TNF family cytokines, including BAFF and APRIL, through the JAK-STAT pathway, initiating inflammatory responses. Autoreactive B cells, stimulated by BAFF and APRIL via BAFFR and TACI, and the Bruton’s tyrosine kinase (BTK) pathway, produce autoantibodies, with CD19 and CD20 contributing to B cell activation. These autoantibodies form immune complexes that sustain adaptive immune responses. B cells present antigens to T_h_ cells via MHC-II and TCR-CD4 interactions, along with CD40-CD40L signaling. Activated T_h_ cells secrete IL-12, promoting T_h_1 differentiation and IFN-γ production, which further enhances BAFF expression—creating a pathogenic feedback loop central to pSS. All one-way arrows indicate activation and forward progression along the pathway, bidirectional arrows represent interactions between components, and truncated lines indicate inhibition.

SS is a multifactorial autoimmune disease characterized by immune dysregulation, with elevated proinflammatory cytokines and autoantibodies driving chronic immune activation and glandular damage [106,107]. Disease onset is primarily mediated by innate immune overactivation, particularly through IFN-I signaling. Isolated and activated pDCs produce IFN-I, BAFF, and APRIL via the JAK–STAT pathway (Figure 6), further stimulating macrophages, NK cells, and CD8^+^ T_ctx_ cells [108,109]. Tissue damage activates TLRs, rapidly inducing IFN-I and initiating proinflammatory cascades [110].

As the disease progresses, adaptive immunity sustains chronic inflammation through (1) autoreactive B cell activation and autoantibody production, (2) B cell–mediated T cell activation and cytokine release, and (3) lymphocyte infiltration of exocrine glands. IFN-I promotes BAFF production, enhancing B cell survival and activation. Stimulated by BCR, BAFF/APRIL-TACI, and TLR signaling, B cells produce pathogenic autoantibodies, notably anti-SSA/Ro and anti-SSB/La, contributing to glandular dysfunction [111].

Autoreactive B cells also act as APCs, engaging CD4^+^ T_h_ cells via MHC-II and CD40–CD40L, promoting proinflammatory cytokine release (e.g., IFN-γ, IL-1, IL-6, TNF-α) and skewing T cell polarization toward T_h_1 and T_h_17 phenotypes while reducing T_h_2 and T_reg_ subsets [112]. IFN-γ further reinforces T_h_1 and CD8^+^ T_ctx_ responses, perpetuating chronic inflammation and reactivating innate pathways through cytokines, BAFF, and immune complexes, establishing a self-sustaining inflammatory loop and characteristic IFN signature [113]. Damaged epithelial cells also contribute as nonprofessional APCs, maintaining immune activation.

SS shows a pronounced female predominance (female-to-male ratio 9:1 to 14:1) [10]. Women typically exhibit stronger humoral responses and higher autoantibody levels, while men present with more severe systemic complications, including vasculitis and pulmonary involvement. Musculoskeletal symptoms (e.g., arthralgia, myalgia) are more frequent in women, whereas men show greater CD8^+^ T_ctx_ infiltration in glandular tissues. The complex interplay between innate and adaptive immunity, along with sex-specific immune variations, presents challenge in SS management. Therapeutic strategies targeting IFN signaling, B-T cell interactions, and cytokine pathways hold promise. Understanding sex-specific immunological differences will be critical for developing more effective, personalized treatments.

Taken together, SS is driven by well-established innate immune activation—particularly pDC-derived IFN-I signaling—triggering downstream inflammatory cascades and B cell hyperactivity. Adaptive immunity further sustains inflammation through autoreactive B cell–T cell interactions, cytokine production, and lymphocytic infiltration of exocrine glands. The role of IFN-I and BAFF/APRIL signaling in promoting B cell survival and autoantibody production is consistently supported by human and animal studies. Emerging evidence highlights the contribution of epithelial cells as nonprofessional APCs and the formation of a self-sustaining inflammatory loop involving IFN-γ and TLR signaling. Pronounced sex differences—stronger humoral responses in females versus greater glandular CD8^+^ T cell infiltration in males—suggest underlying hormonal and immunological influences, though mechanistic clarity is still developing. Overall, while core pathogenic pathways are well-established, several mechanisms remain emerging and warrant further investigation for targeted, sex-informed therapies.

## 5. Sex-Specific Immune Mechanisms in Autoimmune Diseases

The female predominance in SLE and pSS arises from a complex interplay of sex-linked genetic, epigenetic, hormonal, and environmental factors. Key immune-related genes on the X chromosome can escape XCI in females, leading to heightened immune activation. Dysregulated expression of X-linked or autosomal microRNAs (miRs) and histone modifiers further contributes to sex-biased immune activation and tolerance. Estrogens enhance B and T cell activity and IFN-I signaling, whereas androgens exert immunosuppressive effects. These hormones also regulate transcription factors, epigenetic modifiers, and miR expression, influencing cytokine production and immune cell survival. Environmental factors—including viral infections, gut microbiota, environmental chemicals and pollutants, and lifestyle factors—amplify these sex-specific immune pathways. This section highlights the interplay of genetic, hormonal, and environmental factors that drives sex-based differences in immune regulation and disease susceptibility.

### 5.1. Sex-Linked Genetic Factors

#### 5.1.1. Escape from XCI

Sex-based disparities in autoimmune disease prevalence persist even in hormone-independent contexts, such as juvenile rheumatic diseases and postmenopausal women, underscoring a critical role for sex chromosomes in disease pathogenesis [84,114,115]. Comparable hormone levels in prepubescent boys and girls with SLE or SS further support the contribution of X chromosome–linked genetic susceptibility [116].

**Figure 7:**
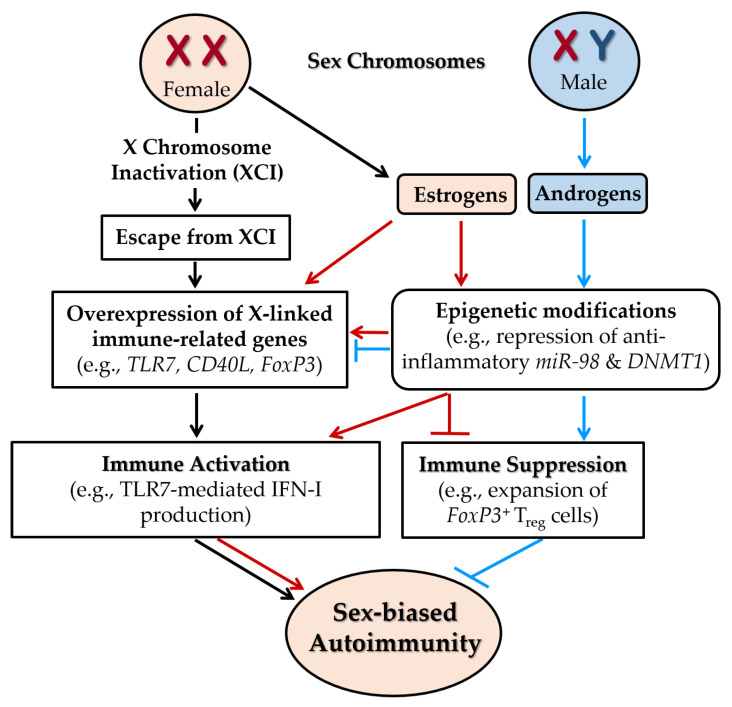
Sex-linked genetic factors contributing to female-biased autoimmunity. Escape from X chromosome inactivation (XCI) in females results in the biallelic expression of immune-related genes such as *TLR7*, *CD40L*, and *FoxP3*, thereby amplifying immune activation. The presence of two X chromosomes also promotes elevated expression of female sex hormones, particularly estrogens. Estrogen signaling further upregulates immune-related gene expression and enhances immune responses, such as TLR7-mediated IFN-I production. Estrogens also exacerbate autoimmunity by inducing epigenetic modifications, including repression of anti-inflammatory microRNAs (e.g., miR-98 in B cells) and inhibition of DNA methyltransferase 1 (DNMT1), which leads to DNA hypomethylation and heightened inflammatory gene expression. In contrast, androgens exert predominantly immunosuppressive effects by promoting DNA methylation to silence proinflammatory genes and by promoting the expansion of *FoxP3^+^* T_reg_ cells. Together, X-linked gene dosage imbalance and hormone-driven epigenetic dysregulation contribute to the increased susceptibility of females to autoimmune diseases. All one-way arrows indicate activation and forward progression along the pathway, while truncated lines indicate inhibition. Arrows and truncated lines are highlighted in black for XCI-dependent pathways, in red for estrogen-dependent pathways, and in blue for androgen-dependent pathways.

Females possess two X chromosomes, while males have one. To maintain dosage compensation, one X chromosome undergoes random inactivation during early embryogenesis in females [117]. However, 20–30% of X-linked genes escape from XCI, leading to functional gene dosage imbalances [116,118,119]. As the X chromosome carries significantly more (11-fold) immune-related genes than the Y chromosome, this escape amplifies immune-regulatory gene expression, thereby increasing autoimmune susceptibility in females [84,120,121] (Figure 7). Clinical observations support this: SLE and SS are rare in Turner syndrome (45,X) but markedly increased (14-fold) in Klinefelter syndrome (47,XXY) and 47,XXX females [122]. Single-cell analyses show biallelic expression of *TLR7* in pDCs, B cells, and monocytes from 46,XX women and 47,XXY Klinefelter males due to skewed XCI [47].

XCI is regulated by the long non-coding RNA Xist, which recruits silencing complexes to epigenetically repress one X chromosome [123]. Disruptions in *Xist* expression or associated protein complexes, observed in thymocytes and peripheral T cells of SLE patients, result in incomplete XCI and reactivation of X-linked immune genes [124]. Together, skewed XCI, gene dosage imbalance, and epigenetic dysregulation contribute significantly to the female predominance in autoimmune diseases [84,125,126].

Collectively, escape from XCI contributes significantly to the female bias in autoimmune diseases, independent of hormonal influences. Strong evidence from genetic, clinical, and single-cell studies shows that biallelic expression of immune-related genes, such as TLR7, due to incomplete or skewed XCI, amplifies immune activation in females. Observations from sex chromosome aneuploidy syndromes (e.g., low autoimmunity in Turner syndrome, high in Klinefelter and 47,XXX) further support this gene dosage effect. Disruptions in *Xist*-mediated silencing have been identified in T cells from SLE patients, suggesting epigenetic dysregulation as a mechanistic contributor. While the dosage imbalance of X-linked immune genes is well-supported, emerging data continue to clarify the specific roles of epigenetic regulators and escapee gene networks in autoimmune pathogenesis.

#### 5.1.2. Immune-Associated Genes Escaping XCI

A subset of immune-related genes on the X chromosome escapes XCI, resulting in biallelic expression in females and individuals with Klinefelter syndrome [65,91,116,127,128,129,130,131,132]. Key genes include *TLR7*, *TLR8*, *CD40L*, *CXCR3*, *IRAK1*, *BTK*, *FoxP3*, *CXorf21*, and *CYBB* (Table 1).

The overexpression of *TLR7* and *TLR8*, encoding endosomal pattern recognition receptors, enhances IFN-I signaling and proinflammatory cytokine production, particularly in pDCs, driving upregulation of IFN-stimulated genes and predisposing females to SLE and related autoimmune diseases [47,125,133]. These receptors also promote B cell activation, class-switching, and autoantibody production, central to SLE pathogenesis [134,135]. Notably, TLR7-mediated IFN-I production is amplified by estrogen signaling, highlighting a convergence of genetic and hormonal effects (Figure 7).

*CD40L*, expressed on activated T cells, promotes pathogenic T_h_ cell responses and B cell activation. Elevated *CXCR3* and *IRAK1* enhances T cell trafficking and innate immune signaling, while *BTK* escape supports autoreactive B cell survival. Dysregulation of *FoxP3*, critical for T_reg_ development and function, impairs peripheral tolerance. CXorf21 (TASL) amplifies IFN-I responses in pDCs and monocytes. *CYBB* encodes a NOX2 subunit essential for reactive oxygen species (ROS) production in phagocytes.

X-linked cytokine receptors (e.g., *IL13RA1/2*, *IL2RG*, *IL9R*) contribute to lymphocyte development and sex-biased immune regulation. Epigenetic regulators *KDM6A* (UTX: ubiquitously transcribed tetratricopeptide repeat, X chromosome) and *KDM5C* (JARID1C), which modulate histone marks, are active in females and associated with increased autoimmune risk [116,136]. *KDM6A*, in particular, enhances NK cell function, as evidenced by reduced IFN-γ production in male and *KDM6A*-deficient female NK cells [137]. These findings underscore the critical role of X–linked gene dosage in shaping immune responses and driving female-biased autoimmunity [138]. Further research into cell type–specific expression and the functional impact of XCI escape genes is essential for advancing precision medicine in autoimmune diseases.

A growing body of evidence supports the role of X-linked immune-related genes escaping XCI—such as *TLR7*, *CD40L*, *IRAK1*, and *FoxP3*—in promoting female-biased autoimmune susceptibility. These genes contribute to heightened IFN-I signaling, B and T cell hyperactivation, and impaired immune tolerance, with consistent findings across human and animal studies. The convergence of XCI escape with estrogen signaling further amplifies immune responses, particularly in SLE. Epigenetic regulators like *KDM6A* and *KDM5C* also escape XCI and have been linked to sex-specific immune modulation. While many of these mechanisms are well-supported, ongoing research is needed to define their cell-type specificity and downstream functional consequences, particularly in the context of sex-informed therapeutic approaches.

#### 5.1.3. Genetic Variations Across the Genome

Sex differences in immune responses are shaped by a complex interplay of X-linked gene expression, XCI, and genome-wide genetic variations. Immune regulatory gene variants and HLA alleles interact with sex to influence disease susceptibility and progression [33]. Recent GWAS have identified over 300 loci associated with sex-biased immune responses, underscoring the intricate genetic contributions to autoimmunity [139]. Notably, most autoimmune-associated GWAS variants lie in non-coding regions and are believed to regulate gene expression through long non-coding RNAs (lncRNAs), which may interact with sex hormones and environmental triggers to modulate immune responses [140]. However, the mechanisms linking these variants to sex-biased autoimmunity remain poorly understood, highlighting the need for functional validation and integrative genomic studies.

X-linked variants play a particularly significant role in sex-biased autoimmunity. Variants in genes such as *TLR7*, *FoxP3*, *IRAK1*, and *MECP2* are strongly associated with increased SLE risk [141]. A notable gain-of-function variant of *TLR7* (Y264H), localized in its ligand-binding domain, enhances its affinity for guanosine-rich ligands, promoting aberrant activation of innate and adaptive immunity by single-stranded RNAs and leading to spontaneous lupus-like disease in kika model mice [142]. This disease phenotype is reversed by *MyD88* deletion, underscoring the pathogenic role of the TLR7–MyD88 axis [91]. Additionally, *TLR7* polymorphisms such as rs3853839 and rs179019 have been linked to increased transcript levels and heightened SLE susceptibility, although results remain inconsistent across studies [143]. In contrast, *TLR9* appears to exert a protective effect, as its deficiency exacerbates disease severity in animal models [144].

Variants affecting the regulation of TLR7 signaling further contribute to disease risk. Risk variants in *SLC29A3*, which modulates TLR7 ligand export from endosomes, lead to nucleoside accumulation and enhanced TLR7 activation. Specifically, rs780669 has been associated with reduced *SLC29A3a* expression in monocytes from Asian SLE patients [145,146]. Mutations in *FoxP3* are implicated in X-linked immune dysregulation syndromes and increase SLE susceptibility [147]. An X-linked SNP in *CCDC22* (rs2294020) has also been associated with enhanced NF-κB activation and increased SLE risk [148,149].

In addition to X-linked factors, autosomal variants and somatic mutations contribute to autoimmune susceptibility. Gain-of-function variants in the IFN-I pathway, a central pathogenic axis in SLE, have been linked to increased disease risk [150]. Sex hormone receptor gene polymorphisms also modulate disease onset and severity. Notably, HLA class II alleles, particularly *HLA-DR*, *HLA-DQA1*, and *HLA-DQB1*, represent some of the most consistent and robust risk factors for autoimmune diseases, including SLE and SS [29].

Collectively, sex-biased autoimmune susceptibility is shaped by a complex interplay of X-linked gene variants, genome-wide polymorphisms, and non-coding regulatory elements. Robust evidence supports the contribution of X-linked variants—particularly in *TLR7*, *FoxP3*, and *IRAK1*—to heightened immune activation in females, with gain-of-function mutations such as TLR7 Y264H promoting lupus-like disease in mouse models. GWAS have identified hundreds of loci associated with immune traits, many in non-coding regions that likely act through lncRNAs and hormone-sensitive regulatory pathways. While the functional relevance of many variants remains unclear, consistent associations with HLA class II alleles and IFN-I pathway genes underscore well-established genetic risks. Emerging data also highlight roles for autosomal variants, somatic mutations, and *TLR7* regulatory genes such as *SLC29A3*, though some findings are population-specific and require further validation. Overall, while several genetic contributors are well-characterized, many mechanisms remain suggestive and call for integrative, sex-informed functional genomics approaches.

#### 5.1.4. Sex-Biased miRs and Gene Expression

miRs are small non-coding RNAs (19–24 nucleotides) that regulate gene expression post-transcriptionally. Aberrant miR expression has been reported in immune cells, including peripheral blood mononuclear cells (PBMCs) and T cells, from autoimmune disease patients [151,152]. Notably, sex-specific differences in miR profiles, observed both intracellularly and in circulating extracellular vesicles, modulate T and B cell functions and contribute to sex-biased immune regulation [33,153,154]. Dysregulated, sex-biased miRs interact with genetic susceptibility loci, transcription factors, and epigenetic modifiers, playing critical roles in the pathogenesis of sex-specific autoimmunity [155]. Consequently, miRs are emerging as potential diagnostic biomarkers, prognostic indicators, and therapeutic targets in autoimmune diseases [151,152,156].

The X chromosome encodes approximately 118 miRs, compared to only 4 on the Y chromosome, contributing to female-biased miR expression in autoimmunity [157]. Skewed XCI further enhances the expression of X-linked miRs. In diseases such as SLE and SS, miRs including miR-20b, miR-23b, miR-98, and miR-222 are frequently downregulated, while miR-106a, miR-223, miR-224, and others are upregulated, supporting their relevance as disease biomarkers and therapeutic targets [158] (Table 2).

In SLE, reduced miR-23b and miR-98 activate NF-κB and STAT3 pathways, promoting proinflammatory cytokine production and autoimmunity [159]. Restoration of these miRs suppresses inflammation and ameliorate disease phenotypes in experimental models [160]. Estrogen may exacerbate autoimmunity by repressing anti-inflammatory miR-98 in B cells (Figure 7). Additionally, miR-548m is upregulated in SLE PBMCs, suppressing PTEN and activating the PI3K–AKT pathway, thereby enhancing immune cell survival [161]. Inhibition of miR-548m restores PTEN and attenuates disease progression. In LN, downregulation of miR-222 correlates with increased *CFHR5* expression and complement activation, contributing to tissue damage [162]. This mechanism is further supported by evidence that lncRNA MIAT exacerbates inflammation by sponging miR-222, leading to upregulation of *CFHR5* [163].

In pSS, downregulation of miR-125b relieves repression on *PRDM1*, promoting plasma cell differentiation and autoantibody production. Restoring miR-125b via exosomes-mediated delivery suppresses *PRDM1* and reduces plasma cell expansion in experiments [164]. Similarly, decreased miR-506 increases *NFATC1* expression, enhancing CD4^+^ T cell activation and proliferation. Pharmacologic upregulation of miR-506 using fangchinoline mitigates T cell–mediated inflammation [165].

miR-223 shows disease-specific expression pattern—upregulated in CD4^+^ T cells and glandular tissues in pSS, but downregulated in active LN [166]. It regulates T cell migration and suppresses proinflammatory chemokines such as CXCL2 and CCL3 by targeting *S1PR1*. In lupus model mice, miR-223 deficiency worsens nephritis, while in SS, its dysregulation promotes epithelial inflammation and cell death [167]. Elevated miR-223 also correlates with altered B cell subset distributions, implicating X chromosome demethylation in the female-biased lupus susceptibility to lupus [152].

Although X-linked miRs play a prominent role, autosomal miRs also contribute to sex-biased autoimmunity. Some function independently of sex hormones, while others are hormonally regulated. For example, miR-21 (Chr17) enhances proinflammatory cytokine production via activation of X-linked *TLR8* [168]. Additionally, dysregulation of miR-145 (Chr5) and miR-224 (X-linked) modulates T cell apoptosis through the STAT1 and apoptosis inhibitor 5 (API5) pathways, particularly in LN [169]. Both X-linked and autosomal miRs shape sex differences in autoimmune diseases through their complex regulation of immune cell function and inflammatory pathways. Further research into sex-specific miR expression and function will be critical for advancing precision medicine strategies in the diagnosis, prognosis, and treatment of autoimmune diseases.

Taken together, sex-biased miRs play a critical and emerging role in autoimmune pathogenesis by regulating immune cell function, gene expression, and inflammatory pathways. X-linked miRs—such as miR-98, miR-223, and miR-222—are often dysregulated in SLE and pSS, contributing to enhanced cytokine production, T and B cell activation, and tissue damage, with consistent findings across patient samples and experimental models. Estrogen-mediated suppression of anti-inflammatory miRs and XCI escape further amplify female-biased miR expression. While some autosomal miRs, such as miR-21 and miR-145, also modulate immune responses and interact with sex-linked targets, their roles remain under investigation. Overall, the evidence for miR involvement is growing but still emerging, and further functional studies are needed to clarify their sex-specific regulatory roles and therapeutic potential in autoimmunity.

### 5.2. Sex Hormones, Pregnancy, and Autoimmunity

The pronounced female predominance in autoimmune diseases such as SLE and pSS is most evident during reproductive years, implicating sex hormones as critical modulators of disease risk [32,48]. For instance, SLE incidence increases nearly nine-fold in females after puberty (ages 15–45), coinciding with elevated estrogen levels [4,170]. In affected women, fluctuations in estrogen and progesterone during menstrual cycles and pregnancy correlate with disease activity, while androgen levels, including testosterone, are often lower [171].

**Figure 8:**
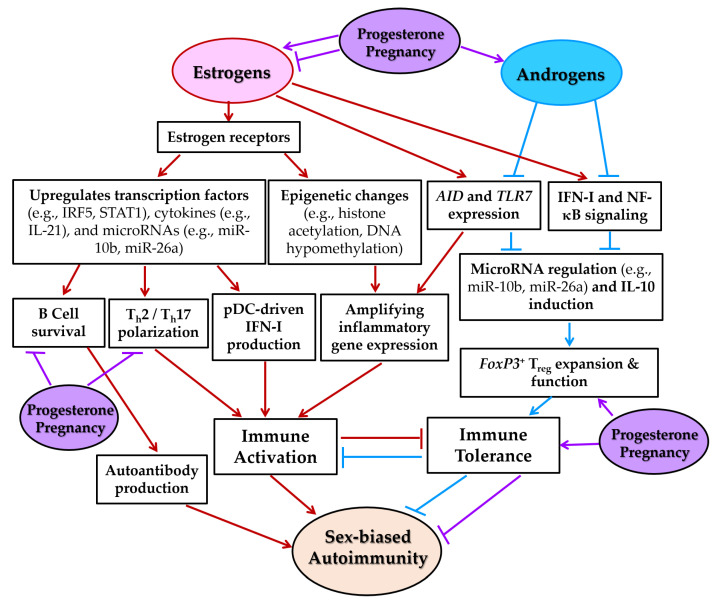
Sex hormone-mediated regulation of immune responses contributing to female-biased autoimmunity. This schematic diagram illustrates how estrogens, progesterone, and androgens differentially modulate immune pathways implicated in the pathogenesis of autoimmune diseases such as SLE and pSS. Estrogens enhance B cell survival, promote T helper 2 (T_h_2) and T_h_17 polarization, and increase pDC-driven IFN-I production by upregulating transcription factors (e.g., *IRF5*, *STAT1*), cytokines (e.g., *IL-21*), and microRNAs (e.g., miR-10b, miR-26a) through estrogen receptor (ER)-mediated signaling. Estrogens also induce epigenetic modifications, including histone acetylation and DNA hypomethylation, that amplify inflammatory gene expression. Progesterone supports T_reg_ function and suppresses T_h_1 and T_h_17 responses, particularly during pregnancy. In contrast, androgens attenuate IFN-I and NF-κB signaling, downregulate activation-induced cytidine deaminase (*AID*) and *TLR7* expression, and promote immune tolerance through expansion of *FoxP3*^+^ T_regs_ and regulation of immunosuppressive factors such as miR-26a and IL-10. Collectively, these hormone-mediated mechanisms contribute to enhanced immune activation and reduced tolerance in females, thereby increasing their susceptibility to autoimmune diseases. All one-way arrows indicate activation and forward progression along the pathway, while truncated lines indicate inhibition. Arrows and truncated lines are highlighted in red for estrogen-dependent pathways, in blue for androgen-dependent pathways, and in purple for progesterone-dependent pathways.

Estrogen promotes immune activation by enhancing B cell responses, increasing autoantibody production, and skewing cytokine profiles toward T_h_2 dominance [125,172] (Figure 8). It also inhibits activation-induced T cell apoptosis by downregulating Fas ligand (*FasL*) and prolongs the survival of activated peripheral T cells. While these effects may support pathogen clearance, they also heighten the risk of autoreactivity. In contrast, androgens exert immunosuppressive effects, suppressing B cell responses, promoting T_h_1/T_h_17 immune profiles, and limiting pathogenic autoantibody production [30,173,174] (Figure 8). This hormonal environment contributes to the lower prevalence and severity of autoimmune diseases in males [121]. Thus, the post-pubertal hormonal milieu in females predisposes them to heightened immune activation and autoimmunity [175,176].

Sex hormones also influence disease onset and severity. In pSS, elevated estrogen levels in middle-aged women are associated with disease development, whereas higher testosterone levels reduce disease severity in animal models [28]. Conversely, the decline of estrogen after menopause promotes glandular apoptosis, increases autoantibody production, and contributes to disease onset. These findings underscore the critical importance of the estrogen-to-testosterone balance in shaping autoimmune disease risk and clinical outcomes.

During pregnancy, rising estrogen and progesterone levels significantly modulate immune responses. Early gestation is characterized by a shift from T_h_1- to T_h_2-dominant immunity, increased T_reg_ cells, and a higher T_reg_/T_h_17 ratio, promoting to maternal–fetal tolerance and reducing inflammation [5]. Progesterone further suppresses T_h_1/T_h_17 responses and B cell activity, potentially mitigating autoimmune flares during pregnancy, particularly in SLE (Figure 8). However, impaired T_reg_ function or pregnancy-related metabolic changes can trigger or exacerbate autoimmunity. Some of these immunological adaptations persist for up to a year postpartum.

Pregnancy also introduces microchimerism—the bidirectional exchange of fetal and maternal cells. Fetal cells can persist in maternal circulation for years, potentially interacting with the maternal immune system and contributing to autoimmune disease development or exacerbation [177]. This may partially explain the higher prevalence of autoimmune diseases in reproductive-aged women. However, pregnancy’s effects on disease course vary: some conditions, such as RA, often improve during gestation, while others, including SLE, may worsen or flare [178].

Collectively, sex hormones are well-established contributors to the female predominance in autoimmune diseases such as SLE and pSS. Elevated estrogen levels during reproductive years enhance B cell activation, cytokine production, and T cell survival, promoting autoreactivity, while androgens exert immunosuppressive effects that limit disease severity in males. Hormonal fluctuations during the menstrual cycle, pregnancy, and menopause influence disease onset and activity, with pregnancy-associated shifts toward T_h_2 and T_reg_ dominance offering transient protection in some cases. However, factors such as impaired T_reg_ function and fetal microchimerism may contribute to postpartum flares or long-term immune dysregulation. Overall, strong clinical and experimental evidence supports sex hormones as key modulators of autoimmune risk and course, with pregnancy introducing additional, complex immunological dynamics.

### 5.3. Sex Hormone-Dependent Mechanisms of Immune Regulation

Sex hormones regulate immune responses through four key mechanisms: (1) modulation of transcription factor activity, (2) amplification of cytokine signaling pathways, (3) induction of epigenetic modifications, and (4) interaction with environmental factors [121,179]. These processes collectively influence immune cell survival, differentiation, and apoptosis, shaping the immune landscape. Understanding these mechanisms is critical for explaining the female predominance in autoimmune diseases and advancing hormone-based therapeutic strategies for SLE, SS, and other immune-mediated conditions.

#### 5.3.1. Modulation of Transcription Factors

Sex hormones regulate immune function primarily through transcriptional control of key immune-related genes. ERs (ERα and ERβ, which exert opposing effects on immune responses) are expressed in most immune cells [37], act as nuclear transcription factors, binding estrogen response element (ERE) to regulate genes such as *TLRs*, *IRF5*, *IFN-I*, *ILs*, *BAFF*, *UNC93B1*, *S1PR2*, *AIRE*, *AID*, and *SLC15A4* [93,125,180,181] (Table 3). ERs also participate in membrane-initiated signaling, further enhancing immune cell activation [182].

Female pDCs produce more IFN-I than male pDCs upon stimulation [32], which may be partly explained by estrogen-mediated upregulation of *IRF5*, a key risk factor for SLE and SS, thereby enhancing IFN-α and proinflammatory cytokine production. In contrast, ERα deficiency reduces *IRF5* expression and impairs pDC function [32,48]. Moreover, estrogens decrease *AIRE* expression, thereby promoting the survival of autoreactive T cells [183]. Conversely, androgen increases its expression, lowering susceptibility of males to develop autoreactive T cells [31]. Estrogens reduces T_reg_ cell numbers by downregulating *FoxP3* expression [184]. Progesterone exhibits dose-dependent immunomodulatory effects. At physiological levels, it supports T_reg_ function via *FoxP3* and *Ikzf2* (*Helios*) upregulation, promoting maternal–fetal tolerance. Progesterone receptor (PR) deficiency in lupus-prone mice leads to reduced T_reg_ and increased T_fh_ cells [171]. At higher concentrations, progesterone may activate glucocorticoid receptors (GRs) or, under certain conditions, cooperate with estrogen to promote retinoid-acid receptor related orphan receptor (*RORγt*) expression and T_h_17 differentiation, contributing to inflammation [185].

Sex hormones also regulate other transcription factors central to autoimmunity. Estrogen-ER complexes activate STAT1 and NF-κB, promoting proinflammatory cytokine production and sustaining the IFN signature in SLE [171,186]. Estrogen additionally induces *HoxC4*, facilitating immunoglobulin class-switch recombination and autoantibody production [186]. In contrast, testosterone and progesterone tend to suppress STAT1, NF-κB, and HoxC4 activity, reducing inflammatory responses and autoimmunity [182,186,187].

Collectively, sex hormones regulate immune responses largely through transcriptional control of immune-related genes via estrogen and progesterone receptors. Estrogen promotes proinflammatory signaling by upregulating *IRF5*, *STAT1*, *NF-κB*, *RORγt*, and *HoxC4*, while downregulating *FoxP3* and *AIRE*, thereby enhancing cytokine production, T cell survival, and autoantibody generation—mechanisms strongly supported in SLE and pSS models. In contrast, androgens and progesterone generally suppress inflammatory transcription factors and promote T_reg_ function through *FoxP3* and *Ikzf2*. These effects are modulated in a dose- and context-dependent manner, with high progesterone levels sometimes contributing to T_h_17-mediated inflammation. Collectively, these hormone-driven regulatory pathways are well-supported and contribute to sex-biased immune activation and loss of tolerance in female-predominant autoimmune diseases.

#### 5.3.2. Amplification of Cytokine Signaling

Aberrant IFN-I signaling is central to the pathogenesis of SLE and SS, with sex hormones serving as critical modulators. In female SLE patients, elevated estradiol correlates with increased expression of IFN-stimulated genes and cytokines such as IL-21 [180]. Estrogen amplifies IFN-I responses by upregulating *TLR7*, *TLR8*, and *TLR9* expression on B cells and DCs, enhancing sensitivity to nucleic acid ligands—particularly during high-estrogen states such as late menstrual phases and pregnancy [171,180]. This effect is mediated through IRF5 and STAT1 signaling, establishing a feed-forward loop that sustains chronic inflammation [32]. In contrast, testosterone suppresses IFN-I production; androgen depletion in lupus-prone mice leads to increased IFN-α secretion and autoantibody production, effects that are further exacerbated by exogenous estrogen administration [32,171].

Sex hormones also exert differential regulation of the NF-κB pathway, a key mediator of inflammatory responses. Estrogen promotes NF-κB activation by suppressing miR-145 and enhancing IκB (inhibitor of NF-κB) kinase-ε (IKKε) activity, thereby increasing proinflammatory cytokine production. In contrast, testosterone and progesterone inhibit NF-κB signaling, reducing the production of TNF-α, IL-6, and T_h_1/T_h_17 cytokines [182,187]. Progesterone, particularly at levels observed during pregnancy, acts through GRs to suppress NF-κB-mediated C-C chemokine ligand 2 (CCL2) expression, supporting immune tolerance [188]. Additionally, PR signaling appears protective in SLE, as PR deficiency exacerbates NF-κB-driven inflammation and worsens disease severity [171].

Beyond IFN-I and NF-κB pathways, sex hormones modulate TLR and BCR signaling, shaping both innate and humoral immune responses. X-linked *TLR7* and *TLR9* escape XCI and are more highly expressed in females [32]. Estrogen further enhances TLR7/8/9 expression [171] and promotes pDC-driven IFN-α production, reinforcing the IFN signature characteristic of SLE [180]. This involves upregulation of UNC93B1 and MyD88, and direct ERα binding to ERE near the *TLR8* locus, further amplifying proinflammatory cytokine production [125,180,189].

In contrast, testosterone downregulates *TLR7* expression and dampens IFN-I responses, providing a protective effect against autoimmunity in males. Experimental evidence shows that castration of lupus-prone mice followed by TLR stimulation induces lupus-like pathology, underscoring the protective role of androgens [48,171]. Sex-specific TLR responses also contribute to distinct autoantibody profiles: estrogen promotes anti-RNP/Sm autoantibodies via TLR7, whereas males predominantly produce anti-dsDNA antibodies through TLR9 activation [32].

In SS, estrogen initially protects glandular epithelial cells but sustained exposure maintains chronic TLR activation and inflammation [48]. Estrogen also promotes autoreactive B cell survival by upregulating *CD22*, *SHP-1*, and *Bcl-2* expression [32]. Conversely, testosterone reduces B cell development and BAFF levels, limiting autoreactive B cell expansion [171]. Although less well characterized, progesterone appears to promote T_h_2 responses and enhance T_reg_ activity, particularly during pregnancy, by increasing IL-10 production and suppressing TLR-induced inflammation [48].

Taken together, sex hormones critically modulate cytokine signaling pathways central to SLE and SS pathogenesis. Estrogen amplifies IFN-I and NF-κB signaling by upregulating *TLR7/8/9* and activating *IRF5*, *STAT1*, and *IKKε*, promoting chronic inflammation and autoantibody production—particularly during high-estrogen states like pregnancy. In contrast, androgens suppress IFN-I and NF-κB pathways and reduce B cell activity, providing protective effects against autoimmunity in males. Progesterone supports immune tolerance through T_reg_ promotion and NF-κB suppression, especially during pregnancy. These hormone-driven differences are strongly supported by both experimental and clinical studies, highlighting a feed-forward loop in females that sustains cytokine-driven inflammation and shapes sex-specific autoantibody profiles.

#### 5.3.3. Induction of Epigenetic Changes

Sex hormones critically influence epigenetic modifications—including DNA methylation, histone modifications, and non-coding RNA regulation—that shape immune gene expression and contribute to sex-based differences in autoimmune susceptibility. In SLE and SS, estrogens promote DNA hypomethylation by inhibiting DNA methyltransferase 1 (DNMT1), leading to overexpression of inflammatory genes in CD4^+^ T cells [32,171] (Figure 8). The *ESR1* gene (encoding ERα) itself becomes demethylated in SLE T cells, creating a self-amplifying loop of heightened estrogen sensitivity. In contrast, testosterone enhances DNA methylation and silences proinflammatory genes, partly by promoting the development of *FoxP3^+^* T_reg_ cells [171]. Progesterone similarly supports T_reg_ expansion and limits T_fh_ cell differentiation. Supporting this, studies in transgender individuals show that estrogen and anti-androgen therapy reduce DNA methylation at proinflammatory loci, whereas testosterone increases it [190]. These hormone-driven epigenetic changes may underlie long-term, sex-specific immune programming [191,192].

Epigenetic dysregulation also directly contributes to autoimmune disease pathogenesis. In SLE, demethylation of X-linked genes such as *CD40L*, *CXCR3*, and *OGT* in CD4^+^ T cells promotes their overexpression, contributing to the female-biased disease phenotype [136,193,194]. Similarly, in diseases such as Takayasu arteritis and psoriatic arthritis, altered DNA methylation in CD8^+^ and γδ T cells affects the expression of key inflammatory genes (*IL1RN*, *IL10*, *IL27*, *IL32*) and components of the TCR signaling cascade [195,196,197].

Non-coding RNAs also play a vital role in sex-biased epigenetic regulation. LncRNAs, including Xist, regulate DNA and histone modifications to maintain XCI. Dysregulated *Xist* expression in female SLE T cells may result in reactivation of X-linked immune genes [198,199]. Additionally, m6A RNA methylation influences T cell development, RNA stability, and alternative splicing, playing an important role in immune homeostasis [200,201].

Estrogen-bound ERα further promotes epigenetic reprogramming by recruiting histone acetyltransferases (HATs) such as p300/CBP, increasing histone acetylation at cytokine gene loci and enhancing inflammatory gene expression [186]. Estrogen also enhances *AICDA* expression, sustaining AID activity and promoting autoantibody production [182,202,203]. Moreover, estrogen inhibits histone deacetylases (HDACs), further sustaining AID expression and autoantibody production [186]. AID interacts with UBN1, a component of the HIRA histone chaperon complex that regulates chromatin structure [181]. In contrast, HDAC inhibitors reduce B cell differentiation and disease activity in lupus-prone mice, indicating their therapeutic potential [204].

Conversely, androgens promote immune tolerance by recruiting co-repressors to condense chromatin and suppress inflammatory genes. AR binding at the *FoxP3* locus alters histone acetylation, enhancing T_reg_ differentiation [171]. AR deficiency leads to increased BAFF levels and B cell hyperactivation, further highlighting its immunoregulatory role.

Incomplete XCI of histone demethylases such as *KDM6A* (UTX) and *KDM5C* amplifies immune gene expression in females. Depletion of *KDM6A* reduces inflammatory cytokine production and tissue damage, underscoring its role in female-biased autoimmunity and its potential as a therapeutic target [32,204].

Finally, environmental exposures—including infections, dietary factors, and xenobiotic agents—interact with sex hormones to modulate epigenetic regulation and influence autoimmune risk [33]. Understanding these complex interactions is critical for identifying sex-specific biomarkers and developing targeted therapies for autoimmune diseases.

Taken together, sex hormones strongly influence epigenetic modifications that shape immune responses and contribute to sex-biased autoimmunity. Estrogen promotes DNA hypomethylation, histone acetylation, and sustained expression of proinflammatory genes and autoantibody-related factors such as AID, especially in SLE and SS. In contrast, androgens enhance DNA methylation and support T_reg_ development via chromatin remodeling. Dysregulated expression of X-linked genes, lncRNAs (e.g., Xist), and histone modifiers like KDM6A further amplifies immune gene expression in females. These mechanisms are supported by experimental, clinical, and cross-sex hormone studies, and are modulated by environmental factors. Collectively, this growing body of evidence highlights hormone-driven epigenetic programming as a critical contributor to female-predominant autoimmunity and a promising target for sex-specific therapeutic strategies.

#### 5.3.4. Regulation of miR Expression

Sex hormones regulate immune gene expression at the post-transcriptional level by modulating miR expression through receptor-mediated mechanisms, thereby shaping immune cell function [32,186,205,206,207]. In SLE, estrogen increases disease-associated miRs in castrated mice, suggesting a pathogenic role for estrogen-regulated miRs in female-biased autoimmunity [125]. Notably, many of these miRs are encoded on autosomes rather than the X chromosome.

Estrogen promotes immune activation by suppressing anti-inflammatory miRs in B cells, including let-7e-5p, miR-98-5p, and miR-145a-5p, leading to increased IKKε expression and enhanced IFN-I signaling [171,187]. Estrogen also downregulates miR-26a, a negative regulator of *AICDA* (a gene of AID), thereby promoting class-switch recombination, somatic hypermutation, and autoantibody production by autoreactive B cells [186]. In T cells, estrogen upregulates miR-10b-5p, inhibiting *SRSF1* and skewing cytokine expression toward proinflammatory profiles [171].

In contrast, androgens promote immune tolerance by enhancing IL-10 production and expanding *FoxP3^+^* T_regs_ [187]. Dihydrotestosterone upregulates miR-26a, reducing *AICDA* expression and limiting B cell activation [186]. These effects contribute to the immunosuppressive influence of androgens and the lower incidence of autoimmunity in males.

Both estradiol and progesterone also promote IL-17A production by inhibiting *let-7f* and increasing *IL-23R* expression, facilitating T_h_17 differentiation and inflammation [185]. These hormone-regulated miR networks add an important layer of control over immune responses, linking hormonal signaling to key effector pathways in autoimmunity. Table 4 summarizes the chromosomal locations, hormone-regulated expression changes, target genes, and immunological functions of key miRs implicated in SLE and related diseases.

Taken together, sex hormones regulate immune responses at the post-transcriptional level by modulating miR expression, thereby shaping immune cell function and contributing to sex-biased autoimmunity. Estrogen suppresses anti-inflammatory miRs (e.g., let-7e, miR-98, miR-145, miR-26a) and upregulates proinflammatory ones (e.g., miR-10b), promoting IFN-I signaling, AID expression, and autoantibody production—key features in SLE pathogenesis. In contrast, androgens exert immunosuppressive effects by enhancing miRs that support T_reg_ expansion and downregulate *AICDA*, thereby limiting B cell activation. Progesterone also contributes to T_h_17 polarization via miR-mediated pathways. These hormone-regulated miR networks are supported by both murine and human studies and represent a growing area of emerging evidence linking hormonal cues to immune dysregulation in autoimmune diseases.

### 5.4. Interplay with Environmental Factors

**Figure 9:**
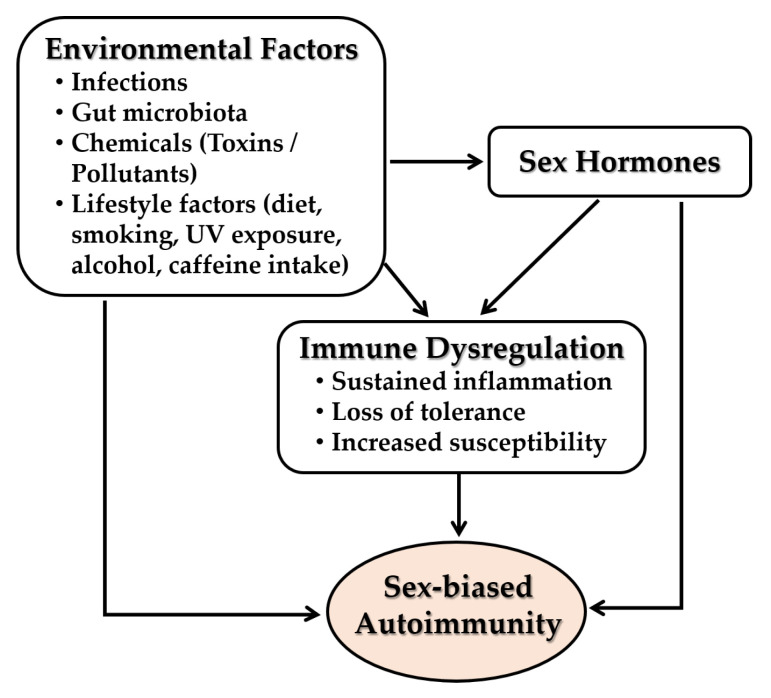
Interactions between environmental factors, sex hormones, and immune dysregulation in the pathogenesis of sex-biased autoimmune diseases: Environmental triggers—including infections, alterations in gut microbiota, exposure to chemicals or pollutants, and lifestyle factors—can disrupt immune homeostasis and promote immune dysregulation. These effects are further modulated by sex hormones, which influence immune gene expression and epigenetic programming in a sex-specific manner. The interplay between environmental insults and hormone-mediated regulation contributes to sustained inflammation, breakdown of immune tolerance, and heightened susceptibility to autoimmune diseases. All arrows indicate activation and forward progression along the pathway.

Emerging evidence highlights the contribution of environmental factors such as infections, gut microbiota, environmental chemicals and pollutants, and lifestyle behaviors such as diet, smoking, UV exposure, alcohol and caffeine intake, physical activity, and circadian habits to immune dysregulation and the pathogenesis of autoimmune diseases [208] (Figure 9). Major autoimmune risk may be attributable to gene-environment interactions. Sex hormones further modulate these effects through sex-specific interactions with environmental insults. These interactions alter hormone-responsive immune gene expression and epigenetic landscapes, increasing disease susceptibility and severity in females.

#### 5.4.1. Infections

Infectious agents—including viruses such as Epstein–Barr virus (EBV), cytomegalovirus (CMV), human immunodeficiency virus (HIV), parvovirus B19, influenza, and SARS-CoV-2) and bacterial components, are major environmental triggers of SLE and pSS [1,209,210,211,212,213]. These pathogens contribute to disease onset and flares by activating innate immunity, inducing IFN-I, recruiting autoreactive lymphocytes, and promoting epitope spreading, molecular mimicry, and bystander activation [214,215,216]. Any robust immune stimulus could theoretically tip the balance in susceptible individuals. Particularly, intercurrent infections often stimulate the IFN-I pathway, T cell activation, and the formation of neutrophil extracellular traps (NETs), precipitating SLE flares [209,217,218,219].

EBV shows the strongest association with SLE and pSS [1,220,221]. SLE patients exhibit higher EBV seropositivity and antibody titers, with elevated (up to 100-fold) latent membrane protein (LMP1) expression in B cells, indicating latent viral reactivation [1,222]. EBV drives autoimmunity by activating TLRs via non-coding RNAs and by molecular mimicry—e.g., EBV nuclear antigen 1 (EBNA-1) cross-reacts with Sm autoantigens in lupus, and EBNA-2 shares homology with Ro60 in pSS [221]. EBV DNA is frequently detected in salivary glands of SS patients, with a meta-analysis showing strong serological association with pSS [220]. EBV miRs may impair glandular function by targeting calcium signaling molecules, while its viral IL-10 homolog fosters local immune tolerance and chronic infection in pSS [221]. Although EBV exposure is nearly universal, only individuals with underlying genetic susceptibility—often associated with *HLA-DR* variants and high viral loads—develop autoimmune diseases [223]. Taken together, microbial exposure is an important environmental insult that can trigger nucleic-acid sensing pathways and loss of tolerance in genetically susceptible hosts, potentially initiating or exacerbating SLE and pSS in females.

Sex hormones modulate antiviral responses and may contribute to sex-biased autoimmunity. Estrogen enhances TLR7-IFN-α signaling, amplifying responses to viral stimuli in females [180], whereas testosterone dampens inflammation, reducing SLE flares in males [32].

Despite their immunosuppressive treatment burden, preventive measures like vaccination remain underutilized in SLE, though vaccine-associated autoimmunity appears rare and unsupported at the population level [1,224]. Persistent viral infections serve as chronic immune stimuli in genetically predisposed hosts, driving IFN-I production, immune dysregulation, and loss of tolerance in both SLE and pSS [225].

Collectively, environmental factors—particularly viral infections, microbiota disturbances, pollutants, and lifestyle behaviors—are emerging as critical contributors to immune dysregulation and autoimmunity, especially in genetically susceptible individuals. Among pathogens, EBV shows the strongest association with SLE and pSS through mechanisms such as TLR activation, molecular mimicry, and viral reactivation in target tissues. These effects are amplified in females due to estrogen-enhanced TLR7–IFN-I signaling, while androgens exert protective effects. Although EBV exposure is nearly universal, only individuals with genetic risk alleles (e.g., *HLA-DR*) develop disease, underscoring the importance of gene-environment-hormone interactions. This growing body of evidence supports environmental exposure as a potent trigger of autoimmunity, with sex hormones shaping the nature and severity of the immune response.

#### 5.4.2. Gut Microbiota

The gut microbiota is a key modulator of immune homeostasis and mediates host–environment interactions. Gut dysbiosis contributes to autoimmunity through immune activation, gut barrier disruption, and microbial mimicry [216,226,227,228]. In lupus-prone models, microbiota depletion reduces inflammation predominantly in females, highlighting sex-dependent microbial influences [229]. Estrogen fosters a proinflammatory microbiome, while androgens promote protective profiles [216]. Microbiota transfer studies show male-derived microbiota can protect against T1D and lupus in female mice [48]. Gut microbes also regulate sex hormone levels, influencing disease trajectories [230]. Microbial metabolites interacting with ERs and PPARs further modulate immune responses.

In SLE, female patients exhibit reduced microbial diversity and enrichment of pathobionts like *Ruminococcus (Blautia) gnavus* (*RG*), which correlates with disease activity and LN [231]. *RG* strains from SLE patients increase gut permeability and translocate to lymphoid tissues, inducing systemic inflammation, whereas *Lactobacillus* exerts protective effects [232]. Some *RG* antigens cross-react with anti-dsDNA antibodies, exemplifying molecular mimicry [227]. Other gut microbes, such as *Bacteroides* and *Odoribacter*, express autoantigen-mimicking peptides (e.g., Ro60, Sm) that activate IFN-γ/IL-17-producing T cells or autoantibody responses in murine models [227,233,234]. Therapeutic strategies targeting gut integrity or composition (e.g., probiotics, zonulin inhibitors) reduce microbial translocation and lupus activity in animal models [227,231]. Thus, dysbiosis acts as an environmental amplifier of SLE by disrupting mucosal tolerance and promoting systemic autoimmunity.

Emerging data indicate similar microbial disturbances in pSS. Patients show decreased microbial richness and lower levels of beneficial commensals (e.g., *Bifidobacterium*, *Agathobacter*), alongside increased *Prevotella*, which is linked to T_h_17-driven inflammation and dry eye severity [235]. Gut microbiota from pSS patients can reduce T_regs_ and enhance T_h_17 responses in recipient mice [236], implicating dysbiosis in systemic immune dysregulation. Additionally, gut-derived metabolites and translocated microbial products may trigger innate immune pathways and cytokine release that affect glandular function. Although mechanistic studies are ongoing, molecular mimicry involving glandular autoantigens (e.g., Ro/La, muscarinic receptors) is a plausible link. Collectively, these findings suggest that gut microbiota imbalances in SLE and pSS contribute to immune dysfunction and symptom exacerbation, representing a promising target for therapeutic intervention of sex-biased autoimmune diseases.

Taken together, gut microbiota play a critical role in modulating immune homeostasis, with dysbiosis emerging as a sex-influenced amplifier of autoimmunity in SLE and pSS. In lupus models, estrogen promotes proinflammatory microbial profiles, while androgens confer protection—evidenced by microbiota transfer studies showing male-derived microbiota reduce disease in female mice. In SLE, pathobionts like *RG* correlate with LN and drive inflammation via molecular mimicry and gut barrier disruption. Similarly, in pSS, dysbiosis is linked to reduced T_regs_, increased T_h_17 responses, and glandular dysfunction. Although mechanistic data are still emerging, these findings highlight gut microbiota as both a modulator of sex hormone activity and a promising therapeutic target in female-biased autoimmune diseases.

#### 5.4.3. Environmental Chemicals and Pollutants

Environmental exposures are critical contributors to systemic autoimmunity. Low concordance rates for SLE among monozygotic twins (~24%), geographic clustering near polluted areas, and urban–rural differences underscore the role of external factors [191,237]. Chemical agents—including pesticides, bisphenol A (BPA), silica, air pollutants, and heavy metals—are linked to increased SLE risk [208,238]. In particular, BPA acts as an endocrine disruptor, promoting autoimmunity via estrogenic signaling [239], while silica and asbestos induce oxidative stress, T_reg_ depletion, and proinflammatory cytokine production [240,241]. Exposure to other xenobiotics, such as drugs, cosmetics, food additives, plant constituents, and environmental pollutants induces oxidative stress and mitochondrial DNA release, activating innate immune pathways [33]. Sex hormones modulate susceptibility to these environmental insults. Estrogen amplifies immune responses to oxidative and apoptotic stress, lowering the threshold for autoimmunity, while testosterone offers partial protection unless exposure is severe [32,33].

Silica exposure, particularly in occupational settings (e.g., miners, sandblasters), is a well-established SLE risk factor [238]. Inhaled silica particles trigger cell death and innate immune activation, promoting IFN-I responses and ectopic lymphoid formation in lungs of lupus-prone mice, accelerating loss of tolerance and autoantibody production [1,242]. Another class of harmful exposures is heavy metals. Mercury, in particular, is a proinflammatory immunotoxicant that can modify host proteins (creating neoantigens), disrupt redox homeostasis, and activate autoreactive B cells, contributing to lupus-like features in both humans and animal models.

Agricultural exposures are particularly relevant. High cumulative pesticide exposure significantly increases the risk of SLE or pSS overtime, with herbicides like metribuzin linked to more than fivefold elevated risk in older individuals [238]. These findings suggest certain pesticides can act as triggers, potentially via immune-adjuvant effects or direct toxicity to lymphocytes. However, not all chemicals have the same impact and that some correlations may reflect complex behavioral or exposure patterns. Notably, early-life farm residence has been associated with reduced risk, supporting the hygiene hypothesis.

Organic solvents and pollutants are another concern. Occupational contact with solvents (such as trichloroethylene or benzene derivatives) has been associated with SLE development, possibly through mechanisms of oxidative stress and epigenetic changes in immune cells [238]. Air pollutants, including particulate matter 2.5 (PM_2.5_), nitrogen dioxide (NO_2_), and polycyclic aromatic hydrocarbons (PAHs), have been associated with increased lupus incidence and disease flares [243,244,245]. Mechanistically, inhaled PM alter DNA methylation in immune cells, upregulate IFN-responsive genes, and promote ongoing disease by epigenetic reprogramming of immune responses [246]. NO_2_ exposure is linked to higher hospitalization rates and mortality in SLE patients, likely through oxidative stress-induced immune dysregulation [243]. While NO_2_ affects both sexes, sex-specific susceptibilities remain to be fully clarified. PAH, a component of smoke and smog, can activate aryl hydrocarbon receptors on immune cells, skewing T cell differentiation and enhancing autoreactive B cell survival.

While fewer studies have examined chemical exposures in pSS, shared environmental risk factors are likely [238]. Gut and lung immune activation by xenobiotics, including silica and pesticides, may promote glandular autoimmunity. Dysregulated clearance of cellular debris, cytokine production (e.g., IL-1, TNFα), and IFN-I activation underlie a common immunopathogenic axis in both SLE and pSS.

Taken together, environmental chemicals and air pollutants act as potent triggers or amplifiers of autoimmunity, especially in genetically predisposed individuals. Avoidance strategies—such as limiting pesticide use, minimizing air pollution exposure, and using protective equipment—may reduce disease risk and progression.

Taken together, environmental chemicals and pollutants are established contributors to autoimmune risk, particularly in genetically predisposed individuals. Agents such as silica, BPA, pesticides, heavy metals, and air pollutants (e.g., PM_2_._5_, NO_2_, PAHs) induce oxidative stress, epigenetic changes, and innate immune activation, promoting IFN-I signaling and loss of tolerance. Estrogen amplifies these effects, while testosterone offers partial protection, contributing to sex-biased vulnerability. Silica and mercury have robust experimental and epidemiological links to SLE, and cumulative pesticide exposure is associated with increased risk of both SLE and pSS. Although sex-specific mechanisms are still emerging, the consistent link between toxic exposures and immune dysregulation underscores the importance of environmental mitigation in autoimmune disease prevention.

#### 5.4.4. Lifestyle Behaviors

Lifestyle behaviors—such as diet, smoking, UV exposure, alcohol and caffeine intake, physical activity, and circadian habits—profoundly shape autoimmune disease risk and severity [237]. Diet and nutritional status influence immune responses via modulation of inflammation, gut microbiota, and hormone interactions [247,248]. In lupus, high-fat and high-sugar diets that lead to obesity may worsen SLE outcomes through proinflammatory adipokines (e.g., IL-6, TNFα), while fiber-rich, low-carbohydrate diet or Mediterranean diets rich in antioxidants and polyunsaturated fats may attenuate systemic inflammation and disease activity [1]. In pSS, dietary research is limited, but adherence to anti-inflammatory diets correlates with lower disease scores and improved hydration supports symptomatic relief [249]. Adequate hydration and avoidance of diuretic substances (like caffeine or alcohol) are often recommended in pSS to help manage dryness, though these are symptomatic measures. These dietary factors modulate inflammation, gut microbiota composition, and interact with sex hormones [247,248].

Cigarette smoking is a prominent lifestyle factor impacting systemic autoimmunity, though its effects differ between SLE and pSS. In SLE, current smoking exacerbates disease risk and morbidity by inducing DNA adducts, oxidative DNA damage, upregulation of BAFF/BLyS/TNFα/IL-6, activation of IFN-I, increased anti-dsDNA autoantibody production, and NETosis, wherein neutrophils release NETs to trap and neutralize pathogens [1,250,251]. Smokers also show reduced IL-10 and increased disease activity and organ damage. Therefore, smoking cessation is strongly recommended for those at risk of or living with SLE, as it removes a significant proinflammatory stimulus to the immune system [252]. In contrast, some studies suggest current smoking may correlate with lower pSS risk [249], possibly due to nicotine’s immunomodulatory effects or disease-related aversion to smoking. Nonetheless, smoking cessation is universally recommended for immune and general health.

UV exposure, particularly UVB, is a well-established environmental trigger in flares and cutaneous lupus lesions. UVB induces keratinocyte apoptosis, exposing nuclear antigens and promoting IFN-I signaling, IFN-regulated gene expression, and T cell activation [1,253,254]. UV-damaged keratinocytes can externalize autoantigens like Ro/SSA, which then incite autoantibody responses. Photosensitivity affects up to 70% of patients, and UV exposure often precedes flares. Females are more susceptible to UV radiation due to hormonal factors. Sunscreen use has been shown to reduce cutaneous and systemic activity [1]. However, UV also contributes to vitamin D synthesis—often deficient in SLE—necessitating a balance between sun protection and supplementation [255]. In pSS, UV associations are weaker as the primary target organs (glands) are internal. However, may trigger symptoms in Ro/SSA-positive or lupus-overlap patients. Photoprotection is generally advised if patients have such symptoms.

Circadian disruption (e.g., shift work, insufficient sleep) is linked to autoimmunity through hormonal rhythm disturbance and immune imbalance [256]. Sleep deprivation increases IL-6 and TNFα levels and reduces T_reg_ function, leading to a proinflammatory cytokine milieu and loss of self-tolerance, and promoting autoreactivity [257,258]. Estrogen fluctuations during menstrual cycles correlate with autoimmune flares in SLE and SS [171]. Nighttime light exposure suppresses melatonin, potentially amplifying estrogen-driven inflammation. Chronotherapy, aligning treatment with circadian and hormonal cycles, holds promise for improving disease management.

Moderate alcohol and caffeine consumption may exert anti-inflammatory effects, while excessive intake promotes immune dysregulation and autoimmunity [259]. Regular physical activity is protective, likely through anti-inflammatory and metabolic benefits. Collectively, lifestyle factors interact with genetic and hormonal contexts to modulate immune tolerance. In both SLE and pSS, interventions targeting modifiable behaviors—such as diet, smoking, UV protection, and sleep hygiene—offer practical strategies to reduce disease onset and flares. While the precise biological mechanisms are still being explored, maintaining healthy lifestyle habits—no smoking, balanced diet, regular exercise, and sufficient sleep—is generally thought to support immune tolerance and reduce the chances of autoimmune disease onset or flare. One notable nuance is sun exposure: UV light is a lifestyle factor (related to outdoor activity) that can trigger lupus flares (see below), yet moderate sun exposure is also needed for vitamin D synthesis, which has protective immunoregulatory effects. Patients are encouraged to find a balance (using sunscreens and vitamin D supplements as needed) [1].

Taken together, lifestyle factors—such as diet, smoking, UV exposure, physical activity, sleep, and circadian rhythms—significantly influence autoimmune disease risk and severity. In SLE and pSS, proinflammatory diets, smoking, UV radiation, and sleep disruption exacerbate immune activation, while anti-inflammatory diets, regular exercise, and good sleep hygiene are protective. Estrogen interacts with these behaviors, amplifying inflammatory responses to UV and circadian disruption, particularly in females. Although smoking may paradoxically correlate with lower pSS risk in some studies, its overall immune-damaging effects support universal cessation. The role of sun exposure is nuanced—UV can trigger flares but is also essential for vitamin D synthesis, requiring individualized photoprotection strategies. Overall, targeting modifiable lifestyle behaviors represents a practical, evidence-supported avenue for reducing disease onset and flares, especially in the context of sex-based immune differences.

#### 5.4.5. Psychological and Physical Stressors

Psychological stress is a potent environmental insult that modulates immune responses via the hypothalamic–pituitary–adrenal (HPA) axis and neuroimmune circuits. Chronic stress initially elevates cortisol, but immune cells can become resistant over time, leading to unchecked sympathetic activation and increased proinflammatory cytokines [260]. In both SLE and pSS, patients frequently report disease flares following emotional stress or trauma. Post-traumatic stress disorder (PTSD) and major life events are associated with significantly elevated autoimmune risk—doubling the odds of SLE and increasing the risk of pSS [249,261].

Stress alters regulatory immune functions by disrupting T_reg_ activity and promoting inflammatory mediators such as substance P and catecholamines. Neuroendocrine-immune crosstalk may also affect glandular inflammation in pSS by altering salivary gland blood flow and immune cell infiltration. These mechanisms underscore that stress is not merely a secondary factor but an active contributor to disease pathogenesis.

Physical trauma and environmental factors such as extreme cold or injury may act as “second hits” that expose sequestered antigens and initiate autoimmune responses in genetically predisposed individuals. Raynaud’s phenomenon, common in lupus and sometimes in pSS, is often triggered by cold exposure. Infections or surgical trauma have also been reported as preceding events in disease onset.

Air pollutants (e.g., particulate matter) can damage lung tissue, increase antigen exposure, and activate innate immune sensors, thereby promoting systemic autoimmunity. These environmental insults often interact with hormonal and genetic susceptibilities, shaping sex-specific disease trajectories. Stress, trauma, and pollution act through diverse mechanisms—oxidative stress, antigen exposure, neuroendocrine imbalance, and IFN-I pathway activation—lowering the threshold for immune tolerance breakdown.

In summary, psychological and physical stressors are important environmental contributors to SLE and pSS pathogenesis. Chronic stress alters neuroimmune regulation via the HPA axis, leading to cortisol resistance, T_reg_ dysfunction, and elevated proinflammatory cytokines. PTSD and major life events are strongly associated with increased autoimmune risk. Physical trauma, cold exposure, and infections can act as secondary triggers, particularly in genetically predisposed individuals. These stress-related effects often intersect with hormonal and genetic factors, contributing to sex-specific immune dysregulation. Collectively, the evidence supports stress and trauma as active drivers—not just consequences—of autoimmune disease onset and flares, underscoring the need for stress management in preventive and therapeutic strategies.

## 6. Conclusions

Autoimmune diseases arise from inappropriate innate and adaptive immune responses to self-antigens, leading to the loss of self-tolerance and chronic tissue damage. Growing evidence underscores the profound impact of biological sex on immune function and autoimmune pathogenesis. Females not only have a higher prevalence of autoimmune diseases but also experience more severe clinical manifestations and frequent disease flares compared to males. These disparities highlight the need to consider sex as a fundamental biological variable in immunological research, diagnostics, and clinical trial design.

**Figure 10:**
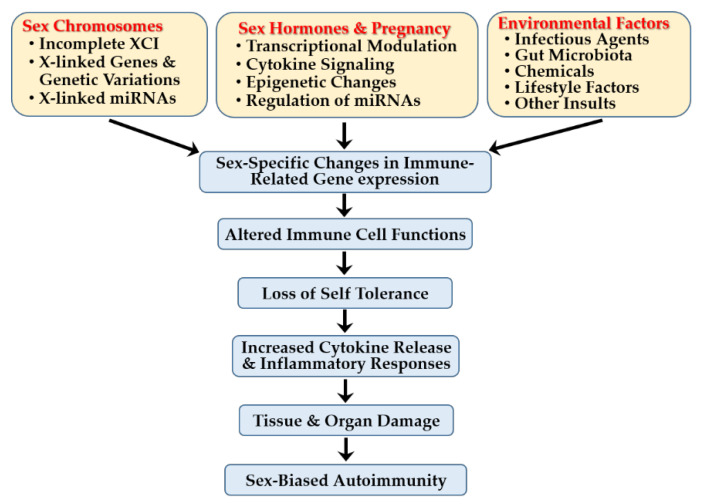
Factors influencing sex-biased autoimmunity.

Sex-specific immune differences result from complex interactions between intrinsic factors—including X chromosome-linked immune gene dosage, genetic variants, miRs, the sex hormone-dependent regulation of transcription, cytokine signaling, epigenetic modifications, and miR expression. These mechanisms shape immune gene expression, immune cell function, cytokine production, self-tolerance, and the activation thresholds of inflammatory pathways (Figure 10). Extrinsic environmental factors—including infections, gut microbiota, chemical exposures, lifestyle behaviors, psychological stress, and circadian disruption—further modulate immune responses in a sex-dependent manner.

Together, these factors drive distinct immune gene expression profiles and contribute to sex-specific disease trajectories. Advancing our understanding of these complex mechanisms will improve risk prediction, enable earlier and more accurate diagnoses, support patient stratification into immunologically homogeneous subgroups, and inform the development of personalized, more effective immunotherapies.

## 7. Future Directions

Future research should focus on elucidating the precise molecular mechanisms underlying the complex interplay among genetic, hormonal, and environmental factors in immune regulation, ideally through integrated multi-omics approaches. Such studies will enhance risk prediction and therapeutic strategies. The development of sex-specific immunotherapies and the consistent inclusion of sex as a biological variable in experimental design, data analysis, and reporting are crucial for advancing personalized prevention and treatment strategies in autoimmunity. Longitudinal, sex-stratified cohort studies and clinical trials are also essential to clarify the influence of sex and hormonal fluctuations on autoimmune disease progression and treatment response.

## Figures and Tables

**Table 1:** X-linked immune genes escaping XCI and contributing to sex differences in autoimmunity.

Gene Symbol	Gene Product	Immunological Function
*BTK*	Burton’s tyrosine kinase	A protein tyrosine kinase that mediates pre-BCR signaling for Ig heavy chain rearrangement, crucial for B cell development and IgE-dependent mast cell activation
*CD40L*	CD40 ligand	A costimulatory molecule on T cells that binds CD40 on APCs, priming pathogenic T_h_ cells, driving immune responses, enabling B-T cell communication and B cell class switching
*CXCR3*	C-X-C motif chemokine receptor 3	A chemokine receptor involved in immune cell trafficking, recruiting killer T cells to sites of inflammation
*CXorf21*	Chromosome X open reading frame 21	A TLR adaptor that interacts with SLC15A4 on the lysosomal membrane
*CYBB*	Cytochrome b-245 beta chain	A component of the NADPH oxidase complex that generates ROS for microbial killing
*FoxP3*	Forkhead box P3	A key regulator of T_reg_ cell development that functions to suppress immune responses
*IL13RA1/2*	Interleukin 13 receptor subunit alpha 1/2	A component of the IL-13 receptor complex that mediates immune regulatory functions
*IL2RG*	Interleukin 2 receptor subunit gamma	A component of the IL-2 receptor essential for T cell development and function
*IL9R*	Interleukin 9 receptor	A component of the IL-9 signaling pathway that regulates diverse immune responses
*IRAK1*	Interleukin 1 receptor associated kinase 1	A protein kinase that mediates IL-1R and TLR signaling to activate NF-κB and MAPK pathways, promoting innate immune responses and inflammation
*KDM6a* *(UTX)*	Lysine demethylase 6a	An enzyme that demethylates H3K27me3 to regulate gene expression, skewing immunity toward inflammation and enhancing NK cell effector function.
*OGT*	O-linked N-acetylglucosamine transferase	An enzyme involved in protein glycosylation that regulates mechanistic target of rapamycin (mTOR) activity and influences diverse cellular processes
*SLC15A4*	Solute carrier family 15 member 4	A proton-coupled amino acid transporter essential for endolysosomal TLR activation and TLR-mediated IFN-I production in innate immune responses
*TLR7*	Toll like receptor 7	A receptor protein that enhances viral RNA sensing and IFN-α production, contributing to female-biased antiviral defense, and promotes ABC accumulation, immune activation, and inflammation
*TLR8*	Toll like receptor 8	A receptor protein involved in TLR signaling via MyD88, sufficient to drive B cell tolerance loss, class-switched autoantibody production, enhanced granulopoiesis, and increased IFN-I production

**Table 2:** Changes in X-linked miRs and functional consequences in autoimmune diseases.

miR	Target Genes	Changes *	Functional Consequences in Autoimmunity
miR-20b	*RELA* (NF-κB subunit), *STAT3*	↓	Its downregulation in SLE T cells lifts repression on *RELA* and *STAT3*, amplifying NF-κB signaling and T_h_17 differentiation, thereby promoting inflammation in lupus pathogenesis.
miR-23b	*TAB2/3* and *IKKα*	↓	Its downregulation in inflamed tissues of SLE, RA, and MS upregulates NF-κB signaling, promoting proinflammatory cytokine production and autoimmunity, while its ectopic expression suppresses inflammation and reduces disease severity in autoimmune models.
miR-92a	*KLF2*, *BCL2L11* (Bim)	↓	Its dysregulation in salivary glands and PBMCs of SS patients may disrupt glandular epithelial cell survival and immune cell homeostasis by altering apoptosis and T cell differentiation.
miR-98	*IL-6*, *FAS* (CD95), *TNF-α*	↓	Its downregulation in SLE PBMCs lifts suppression of *IL-6*, increasing proinflammatory cytokines and STAT3 signaling, while *FAS* upregulation promotes CD4^+^ T cell apoptosis, worsening immune dysregulation and disease activity.
miR-106a	*IL-10*, *SOCS5*	↑	Its upregulation in CD4^+^ T cells and PBMCs from SLE and pSS patients suppresses IL-10, a regulatory cytokine, and SOCS5, enhancing JAK-STAT signaling and promoting T cell activation and inflammation.
miR-125b	*PRDM1* (Blimp-1)	↓	Its downregulation in activated CD4^+^ T cells of SS patients lifts repression on *PRDM1* (Blimp-1: B-lymphocyte-induced maturation protein 1), enhancing plasma cell differentiation and autoantibody production, while its exosomal delivery from salivary gland-derived mesenchymal stem cells suppresses plasma cell formation and restores secretory function.
miR-188	*NFATc2*, *FOXO1*, *CBL*	↑	Its upregulation in PBMCs of SLE, RA, and PA patients suppresses *FOXO1*, impairing T_reg_ differentiation and immune tolerance, while reducing *CBL* expression and dampening TCR signaling in lupus CD4^+^ T_h_ cells, collectively enhancing effector T cell activity.
miR-221 /222 cluster	*CDKN1B* (p27kip1), *ETS1*	↑	Its upregulation in SLE PBMCs downregulates *CDKN1B* and *ETS1*, driving lymphocyte proliferation and plasma cell differentiation, which enhances autoreactive B cell activity and autoantibody production.
miR-222	*CFHR5*	↓	Its downregulation in LN patients increases *CFHR5* expression, overactivating the alternative complement pathway and promoting immune complex–mediated tissue injury.
miR-223	*S1PR1*, *CXCL2*, *CCL3* (in SLE) *ITPR3* (in SS)	↑/↓	Its upregulation in CD4^+^ T cells from SLE patients and in epithelial cells of SS patients suppresses *S1PR1* and chemokines, limiting T cell egress and inflammatory cell recruitment, while downregulating *ITPR3* to impair Ca^2+^ signaling and activate NF-κB, promoting epithelial inflammation. It is also linked to X chromosome demethylation in female lupus predisposition, whereas its deficiency in LN leads to T cell accumulation and exacerbated renal inflammation.
miR-224	*SMAD4*, *HOXD10*, *API5*	↑	Its upregulation in PBMCs and T cells of SLE, SD/SSc, and RA targets *SMAD4*, disrupting TGF-β signaling and promoting fibrosis and tissue dysfunction. It also enhances cell proliferation and migration, downregulates apoptosis inhibitor 5 (*API5*) to facilitate activation-induced cell death in Jurkat and SLE T cells, and upregulates STAT-1, contributing to LN.
miR-361-5P	*VEGFA*, *IL-6R*	↑	Its overexpression in labial salivary glands of SS patients reduces *VEGFA* and *IL-6R* expression, potentially compromising vascular integrity and altering cytokine responses in glandular tissues.
miR-374a	*SOCS1*, *PTEN*, *IL-10*	↑	Its upregulation in inflamed synovium of SLE and RA downregulates *SOCS1* and *PTEN*, activating JAK-STAT and PI3K pathways, thereby promoting cytokine-driven inflammation and lymphocyte survival, and increased susceptibility to SLE with renal involvement.
miR-421	*ATM*, *E2F1*, *PDCD4*	↑	Its upregulation in LN kidney biopsies and RA synovial tissue impairs the DNA damage response by inhibiting ATM and E2F1 in LN renal tissues, suppresses apoptosis-related genes like *PDCD4*, promoting fibroblast-like synoviocyte survival and proliferation in RA.
miR-424	*CCND1*, *CDK6*	↑	Its upregulation induces cell cycle arrest in SLE PBMCs and SS salivary gland epithelial cells by downregulating *CCND1* and *CDK6*, resulting in tissue atrophy and impaired glandular regeneration.
miR-452	*BMI1*, *RAB11A*, *CDKN1B*	↓	Its downregulation derepresses genes that promote T cell proliferation and survival, thereby enhancing autoreactive T cell responses in MS and RA patients.
miR-506	*NFATC1*	↓	Its underexpression lifts repression on *NFATC1*, leading to increased CD4^+^ T cell activation and proliferation in SS patients.
miR-548m	*PTEN*	↑	Its overexpression reduces PTEN expression, resulting in hyperactivation of the PI3K–AKT pathway and promoting immune cell survival and activation in SLE patients.
hsa-miR-503	*BCL2*, *CCND1*, *FGF2*	↓	Its downregulation enhances *BCL2*-mediated survival and *CCND1*-driven proliferation, leading to synovial hyperplasia and joint inflammation in RA, while its expression is elevated in demethylated CD4^+^ T cells from women with lupus following 5-azacytidine treatment.
hsa-miR-545	*RIG-I*, *TP53INP1*, *ZEB2*	↑	Its upregulation, observed in some LN datasets, inhibits *RIG-I*-mediated antiviral responses and regulates *p53*-dependent apoptosis, potentially shifting immune balance away from effective antiviral surveillance.
hsa-let-7f-2	*STAT3*, *IL-13*, *TGFBR1*	↑	Its upregulation in the plasma and salivary glands of SLE and SS patients modulates T_h_2 and T_h_17 differentiation by suppressing *STAT3* and *IL-13*, potentially disrupting effector T cell balance and promoting proinflammatory cytokine production.

* ↑, over-expressed; ↓, under-expressed.

**Table 3:** Immune-related gene products regulated by sex hormones.

Gene Products	Impacts by Sex Hormones
TLRs (Toll-like receptors)	Estrogens and ERα signaling differentially regulate TLR family members, enhancing TLR7/9-mediated IRF5 activation and IFN production in female pDCs, while modulating TLR8 expression independently of IFNs through direct ERα binding to an ERE near the TLR8 locus or indirectly via STAT1-mediated transcriptional activation.
IRF5 (Interferon regulatory factor 5)	Estrogens and ERα signaling upregulate IRF5, a key transcription factor involved in immune responses and a lupus susceptibility factor, leading to IFN-I overproduction and contributing to autoimmune disease progression in SLE.
IFNs (Interferons)	Estrogens enhance IFN-α and IFN-γ production via ERα and TLR7/9-mediated IRF5 activation in pDCs, amplifying cytokine output and innate immunity, thereby contributing to the female bias in autoimmunity. IFN-α upregulates MHC-I, while IFN-γ induces MHC-II and alters proteasome composition, facilitating self-peptide presentation to T cells—processes central to SLE pathogenesis.
ILs (Interleukins)	Estrogens and ERα signaling promote IL-6 expression and inflammation in both mice and humans, while elevated estradiol in SLE patients enhances the secretion of IL-8, IL-18, and IL-23. Estrogen-regulated cytokines such as IL-4, IL-5, and IL-10 support B cell activation and antibody production. Notably, increased IL-6 and IL-10 levels correlate with higher SLE disease activity index (SLEDAI) scores, linking estrogen-driven cytokine expression to disease activity in SLE.
BAFF (B cell activating factor)	Estrogens enhance BAFF production, which supports B cell survival and maturation, leading to elevated antibody levels and potentially influencing thyroid dysfunction in GD.
UNC93B1 (Unc-93 homolog B1)	Estrogens enhance UNC93B1 expression via IFN-α or IFN-γ signaling, with notably higher levels observed in lupus-prone female mice and PBMCs from SLE patients compared to healthy controls.
S1PR2 (Sphingosine-1-phosphate receptor 2)	Estrogens regulate S1PR2 expression, a G protein-coupled receptor, potentially contributing to the female-biased severity of CNS-related autoimmune diseases like MS.
AIRE (Autoimmune regulator)	Estrogens increase methylation of CpG sites in the AIRE promoter, inducing epigenetic silencing of AIRE—a central tolerance regulator controlling tissue-specific antigen expression—thereby enhancing autoimmune susceptibility. In contrast, androgens upregulate AIRE, contributing to sex bias in CNS autoimmune diseases.
AID (Activation-induced cytidine deaminase)	Estrogens promote AID transcription, enhancing somatic hypermutation and class switch recombination in activated B cells—key processes for antibody diversification—likely through AID’s interaction with the chromatin modifier UBN1, a component of the HIRA chaperone complex.
SLC15A4 (Solute carrier family 15 member 4)	Estrogens upregulate SLC15A4 expression, enhancing IFN-I and proinflammatory cytokine production in pDCs, thereby contributing to autoimmune disease progression in SLE and colitis models.
Cathepsin S	Estrogens promote inflammation by activating cathepsin S—a lysosomal acidic protease involved in immune regulation—elevated in the lacrimal glands and tears of female SS murine models., whereas testosterone reduces inflammation and enhances glandular function in SS, potentially through cathepsin S suppression.

**Table 4:** Estrogen-dependent autosomal miRs and their functions in autoimmune diseases.

miR	Location	Change *	Target Gene	Impacts by Sex Hormones
miR-10b	Chr2	↑	*SRSF1*, *MAPK7 (TAK1)*	Estrogens upregulate miR-10b-5p, which inhibits SRSF1 and MAPK7, enhancing NF-κB signaling, T_h_17 differentiation, and proinflammatory cytokine expression in T cells.
miR-26a	Chr3/ Chr12	↓	*AICDA*, *HMGA2*, *COX-2*, *TLR4*, *MALT1*, *HMGA1*	Estrogens suppress miR-26a, enhancing class-switch recombination and autoantibody production, while androgens induce miR-26a to restrain *AICDA* expression and B cell activation. miR-26a downregulation also enhances proinflammatory cytokines via other target genes.
miR-31	Chr9	↓	*RhoA*, *CEACAM1*, *IRF5*, *STAT-1*, *SLC15A4*	Estrogens downregulate miR-31 via TGF-β and NF-κB in SLE T cells, impairing IL-2 production by disrupting NFAT, NF-κB, and AP-1 activity, while increasing CREM and dysregulating CaMK-IV and PP2A. This leads to defective IL-2 signaling, impaired T_reg_ differentiation, and T cell dysfunction.
miR-96	Chr7	↑	*FoxP3*, *RHOA*, *FCGR1*, *IL-2*, *CD138*, *CEACAM1*	Estrogen-induced miR-96 upregulates immune genes such as *FoxP3*, *RHOA*, *FCGR1*, *IL-2*, *CD138*, and *CEACAM1*, influencing SLE susceptibility, onset, clinical heterogeneity, and progression.
miR-127	Chr14	↑	*FoxP3*, *RHOA*, *FCGR1*, *IL-2*, *CD138*, *CEACAM1*	Estrogen-induced miR-127 promote immune and inflammatory responses, contributing to SLE susceptibility, onset, clinical heterogeneity, and progression.
miR-145	Chr5	↓	*STAT1*, *OPG*	Estrogen-mediated miR-145-5p downregulation elevates osteoprotegerin, reducing osteoclast activity and bone resorption, and contributing to joint damage in RA.
miR-145a	Chr5	↓	*ADAM17*, *KLF4*, *SIRT1*	miR-145a targets inflammation- and stress-related genes to suppress immune activation, but estrogen downregulates miR-145a in B cells, promoting immune activation.
miR-146a	Chr5	↑↓	*IRAK1*, *TRAF6*, *IRF5*, *STAT-1*, *SLC15A4*	miR-146a suppresses IRAK1 and TRAF6 translation, serving as a negative regulator of immune activation. Estrogens dysregulate miR-146a in PBMCs and splenocytes of MRL/lpr mice, linking it to epigenetic changes, B cell hyperactivity, and autoantibody production in autoimmunity.
miR-148a	Chr7	↑	*DNA methytransferase 1 (* *DNMT1* *)*	Upregulated miR-148a promotes DNA hypomethylation, contributing to autoimmune disease pathogenesis.
miR-148b	Chr12	↑	*CaMKIIα*, *Gadd45α*, *PTEN*, *Bim*	miR-148b suppresses TLR-induced cytokine and IFN-I production, impairing DC-medicated innate responses. It also promotes DNA hypomethylation and survival of autoreactive B cells, contributing systemic autoimmunity.
miR-155	Chr21	↑	*MAPK*, *INS*, *Wnt*, *NF-κB*, *BIC*, *Pu.1*, *c-Maf*, *c-Fos*, *IFNγRα*, *c-Rel*, *c-Fos*, *Peli1*, *p27kip1*, *KPC1*, *SOCS1*	miR-155 regulates immune cell homeostasis, T_h_1 differentiation, tolerance, and development. It supports B cell maturation, isotype switching, germinal center formation, high-affinity IgG1 production, DC activation, apoptosis, and IL-12 production, promoting autoimmune susceptibility.
miR-183	Chr7	↑	*FoxP3*, *RHOA*, *FCGR1*, *IL-2*, *CD138*, *CEACAM1*	miR-183 modulates immune and inflammatory responses, contributing to susceptibility to SLE and influencing its onset, clinical heterogeneity, and progression.
miR-379	Chr14	↑	*FoxP3*, *RHOA*, *FCGR1*, *IL-2*, *CD138*, *CEACAM1*	miR-379 modulates immune and inflammatory responses, influencing SLE susceptibility, onset, heterogeneity, and progression.
let-7e	Chr19	↓	*SOCS1*, *TLR6*, *TLR9*	let-7e targets SOCS1 and TLR pathway components, regulating cytokine signaling and innate immunity. Estrogens suppress let-7e in B cells enhancing immune activation.
let-7f	Chr9/ ChrX	↓	*IL-23R*, *NLRP3*, *IL-6*, *A20 (TNFAIP3)*	let-7f-5p targets NLRP3, mitigating inflammation in bone marrow-derived mesenchymal stem cells, and also regulates IL-6 and A20, key modulators of inflammatory and NF-κB signaling pathways. Estradiol and progesterone suppress let-7f and upregulate *IL-23R*, enhancing IL-17A production in T_h_17 cells, thereby promoting T_h_17 differentiation and inflammation.

* ↑, over-expressed; ↓, under-expressed.

## Data Availability

Not applicable.

## Abbreviations

The following abbreviations are used in this manuscript:

ABC, age-associated B cell; AID, activation-induced cytidine deaminase; AIRE, autoimmune regulator; anti-dsDNA, antinuclear antibodies against double-stranded DNA; APCs, antigen-presenting cells; AP-1, Activator Protein-1; API5, apoptosis inhibitor 5; APRIL, a proliferation-inducing ligand; BAFF, B cell activating factor; BAFFR, B cell activating factor receptor; BCR, B cell receptor; BPA, bisphenol A; BTK, Bruton’s tyrosine kinase; CCL2, C-C chemokine ligand 2; *CXCR*, C-X-C motif chemokine receptor; DCs, dendritic cells; DNMT1, DNA methyltransferase 1; EBNA, EBV nuclear antigen; EBV, Epstein–Barr virus; ER, estrogen receptor; ERE, estrogen response element; FoxP3, forkhead box P3; GD, Graves’ disease; GR, glucocorticoid receptor; GWAS, Genome-Wide Association Study; HLA, human leukocyte antigen; HPA, hypothalamic–pituitary–adrenal; IFN-I; type I interferon; IKK, IκB (inhibitor of NF-κB) kinase; IL, interleukin; iNKT cell, invariant natural killer T cell; IRAK, interleukin 1 receptor-associated kinase; IRF, interferon regulatory factor; JAK-STAT, Janus kinase-signal transducer and activator of transcription; KDM6A, lysine demethylase 6A; LN, lupus nephritis; lncRNAs, long non-coding RNAs; MAPK, mitogen-activated protein kinase; MHC, major histocompatibility complex; miR, microRNA; MS, multiple sclerosis; MyD88, myeloid differentiation factor 88; NETs, neutrophil extracellular traps; NF-κB, nuclear factor kappa B; NK cell, natural killer cell; NO_2_, nitrogen dioxide; PAH, polycyclic aromatic hydrocarbons; PBMCs, peripheral blood mononuclear cells; pDC, plasmacytoid dendritic cell; PDCD4, a selective protein translation inhibitor; PM, particulate matter; PR, progesterone receptor; pSS, primary Sjögren’s syndrome; PTSD, post-traumatic stress disorder; RA, rheumatoid arthritis; RG, *Ruminococcus (Blautia) gnavus;* ROR, retinoid-acid receptor related orphan receptor; ROS, reactive oxygen species; SD, scleroderma; SLC15A4, endolysosomal solute carrier family 15 member 4; SLE, systemic lupus erythematosus; Sm, Smith protein; snRNP, small nuclear ribonucleoprotein; SOCS1, suppressor of cytokine signaling 1 (a negative regulator of the JAK-STAT pathway); SS, Sjögren’s syndrome; SSA/SSB, Sjogren’s autoantibodies A and B; SSc, systemic sclerosis; TACI, transmembrane activator and calcium moderator and cyclophilin ligand interactor; TAK1, transforming growth factor-β-activated kinase 1; TASL, TLR adaptor interacting with SLC15A4; TCR, T cell receptor; T_ctx_ cells, cytotoxic T cells; T_h_ cells, helper T cells; TGF-β, transforming growth factor-beta; TLR, Toll-like receptor; TNF-α, tumor necrosis factor-alpha; T_reg_ cells, regulatory T cells; UTX, ubiquitously transcribed tetratricopeptide repeat, X chromosome; XCI, X chromosome inactivation.
